# Multiple Tin Compounds Modified Carbon Fibers to Construct Heterogeneous Interfaces for Corrosion Prevention and Electromagnetic Wave Absorption

**DOI:** 10.1007/s40820-024-01527-w

**Published:** 2024-09-27

**Authors:** Zhiqiang Guo, Di Lan, Zirui Jia, Zhenguo Gao, Xuetao Shi, Mukun He, Hua Guo, Guanglei Wu, Pengfei Yin

**Affiliations:** 1 College of Science, Sichuan Agricultural University, Ya’an, 625014 People’s Republic of China; 2 School of Materials Science and Engineering, Hubei University of Automotive Technology, Shiyan, 442002 People’s Republic of China; 3 Institute of Materials for Energy and Environment, State Key Laboratory of Bio-Fibers and Eco-Textiles, College of Materials Science and Engineering, Qingdao University, Qingdao, 266071 People’s Republic of China; 4 Shaanxi Key Laboratory of Macromolecular Science and Technology, School of Chemistry and Chemical Engineering, Northwestern Polytechnical University, Xi’an, Shaanxi 710072 People’s Republic of China

**Keywords:** Electrostatic spinning, Component regulation, Heterogeneous interfaces, Electromagnetic wave absorption, Corrosion protection

## Abstract

Currently, the demand for electromagnetic wave (EMW) absorbing materials with specific functions and capable of withstanding harsh environments is becoming increasingly urgent. Multi-component interface engineering is considered an effective means to achieve high-efficiency EMW absorption. However, interface modulation engineering has not been fully discussed and has great potential in the field of EMW absorption. In this study, multi-component tin compound fiber composites based on carbon fiber (CF) substrate were prepared by electrospinning, hydrothermal synthesis, and high-temperature thermal reduction. By utilizing the different properties of different substances, rich heterogeneous interfaces are constructed. This effectively promotes charge transfer and enhances interfacial polarization and conduction loss. The prepared SnS/SnS_2_/SnO_2_/CF composites with abundant heterogeneous interfaces have and exhibit excellent EMW absorption properties at a loading of 50 wt% in epoxy resin. The minimum reflection loss (RL) is − 46.74 dB and the maximum effective absorption bandwidth is 5.28 GHz. Moreover, SnS/SnS_2_/SnO_2_/CF epoxy composite coatings exhibited long-term corrosion resistance on Q235 steel surfaces. Therefore, this study provides an effective strategy for the design of high-efficiency EMW absorbing materials in complex and harsh environments.

**Supplementary Information:**

The online version contains supplementary material available at 10.1007/s40820-024-01527-w.

## Highlights

- Excellent impedance matching through component modulation engineering.
- Rich heterogeneous interfaces are constructed to realize excellent electromagnetic wave (EMW) absorption performance.
- Long-term corrosion protection and excellent EMW absorption properties.

**Supplementary Information:**

The online version contains supplementary material available at 10.1007/s40820-024-01527-w.

## Graphical abstract

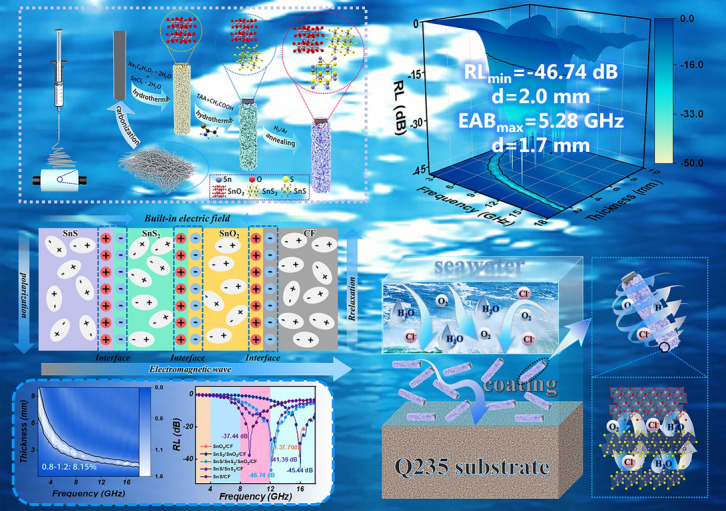

## Introduction

With the advancement of science and technology, the development of electronic products and wireless communication technology has also brought about electromagnetic wave (EMW) pollution problems, which seriously endanger human health [1–7]. Therefore, exploring advanced and multifunctional EMW absorbing materials is the most effective way to solve this problem. The characteristics of excellent EMW absorption materials are light weight, thin thickness, strong absorption capacity and wide effective absorption bandwidth [8, 9]. Expanded graphite (EG), graphene (GR), carbon nanofibers (CNF), conductive polymers, carbon-based biomaterials, metal–organic framework, and their derivatives have attracted much attention as they have the advantages of low density, rich functional groups, and tunable properties [10–15]. However, single-component materials have disadvantages such as poor dielectric properties and impedance mismatch. Therefore, the multi-scale structural design of EMW absorbent materials and construction of multi-component composite materials are effective ways to solve this problem [16–20].

Constructing multi-material systems and forming several heterogeneous interfaces to increase the interface polarization effect is a typical tactic. Multi-component composites could fully utilize the advantages of each component to adjust the impedance matching and enrich the electromagnetic loss mechanism. The electronic and crystal structure asymmetries at non-uniform interfaces lead to lattice distortions, charge mismatches and energy band migration, which dissipate EMW energy [21–25]. As a traditional carbon material, carbon fiber (CF) has a high aspect ratio and electrical conductivity, making it a widely employed component in the EMW absorption field [26–30]. Unfortunately, the impedance matching capability of single-component CF is poor, resulting in a large amount of electromagnetic waves being reflected rather than absorbed during transmission. This limits the prospects for its application in EMW absorption. The introduction of nanocompounds into the composite system can endow the composites with multilayered structure, excellent impedance matching, and enriched EMW loss mechanisms. Semiconductor materials have adjustable conductivity and excellent dielectric properties and can be combined with other materials to form heterogeneous structures [31, 32]. These heterogeneous interfaces can enhance the interface polarization effect and further enhance the EMW absorption performance. Therefore, carbon fibers were composited with semiconductor materials in order to tune the composition, interfaces, and defects of the materials to optimize the EMW absorption properties of the materials [33–36]. Tin dioxide (SnO_2_) semiconductor materials have good chemical stability and unique structural features. It has good electrical conductivity and dielectric properties, and can effectively regulate the dielectric loss of the material. Tin disulfide (SnS_2_) and stannous sulfide (SnS) have a unique layered structure, large layer spacing, high reversible capacity, and excellent electron transport properties [37–40]. These characteristics contribute to the multiple dissipation of EMWs, thus having great application potential in the field of EMW absorption.

Hence, in this work, a variety of stannide-modified CF composites (SnS/SnS_2_/SnO_2_/CF) were constructed. The CF skeleton was first prepared by electrostatic spinning and high-temperature calcination processes. Then, SnS/SnS_2_/SnO_2_/CF with a large number of non-uniform interfaces were successfully prepared by hydrothermal and thermal decomposition reduction processes. The degree of sulfidation and thermal reduction were adjusted by controlling the content of the sulfur source and thermal decomposition temperature, to realize the coexistence of multi-component tin compounds. Multi-component composites have a large number of non-uniform interfaces that can effectively regulate impedance matching. Strong interactions or synergistic effects are generated at the interfaces to realize the interface polarization loss effect and multiple attenuation of EMW, thus enhancing the EMW loss capability. The SnS/SnS_2_/SnO_2_/CF composite achieved minimum reflection loss (RL_min_) = − 46.74 dB at a matched thickness of 2.0 mm and the maximum effective absorption bandwidth (EAB)_max_ = 5.28 GHz at a matched thickness of 1.7 mm. The SnS/SnS_2_/SnO_2_/CF epoxy composite coatings were tested using a three-electrode system, and the results showed that the composite coatings have long-term corrosion resistance. Therefore, this work provides a valuable reference for the preparation of EMW absorbers with thin thickness, lightweight, and excellent absorption performance that can be applied under harsh and complex conditions.

## Experimental Section

### Materials

Tin (II) chloride dihydrate (SnCl_2_·2H_2_O), Trisodium citrate dihydrate (Na_3_C_6_H_5_O_7_·2H_2_O), thioacetamide (TAA, C_2_H_5_NS), acetic acid (CH_3_COOH), N, N-dimethylformamide (DMF, 99%), polyacrylonitrile (PAN, M_w_ = 150,000), anhydrous ethanol (CH_3_CH_2_OH, AR). Deionized water. All chemicals are analytical grade and are used directly without further purification.

### Preparation of Carbon Fiber

First weigh 1 g PAN and add it to a 15 mL beaker. Then add 10 mL DMF and stir for 12 h to obtain PAN spinning solution. Transfer the spinning solution into a 10 mL syringe and attach a 19 G needle. Subsequently, the syringe was placed in the electrostatic spinning device and the parameters were adjusted (Voltage: 19 kV; Injection rate: 0.9 mL h^−1^; Distance between syringe and collection device: 17 cm). The obtained fiber membrane was placed in a vacuum oven at 70 °C to dry overnight. Subsequently, the fiber membrane was placed in a muffle furnace and heated up to 260 °C at 2 °C min^−1^ and held for 2 h. Finally, the pre-oxidized fibers were carbonized in a tube furnace at 900 °C for 3 h, and the heating rate was 5 °C min^−1^ to obtain CF.

### Preparation of SnO_2_/CF

First, 0.3 g of SnCl_2_·2H_2_O and 0.818 g of Na_3_C_6_H_5_O_7_·2H_2_O were weighed and dissolved in a mixed solution of 15 mL of CH_3_CH_2_OH and 15 mL of deionized water and stirred for 1 h. The obtained solution is then transferred to a 50 mL teflon lining and the appropriate amount of CF is added. Subsequently, SnO_2_/CF was obtained by keeping it at 180 °C for 12 h. Finally, wash with water and ethanol and dry overnight in a vacuum oven at 70 °C.

### Preparation of SnS_2_/SnO_2_/CF

First, 0.09 g of TAA was weighed, then 2 mL of CH_3_COOH and 15 mL of deionized water were added and stirred for 40 min to form a homogeneous solution. The solution was then transferred to a 50 mL teflon lining, with the addition of an appropriate amount of SnO_2_/CF, and held at 180 °C for 12 h to obtain SnS_2_/SnO_2_/CF. Finally, it was dried overnight in a vacuum oven at 70 °C. SnS_2_/CF was prepared by adding 0.25 g TAA, and the other steps were consistent with the above method.

### Preparation of SnS/SnS_2_/SnO_2_/CF

SnS_2_/SnO_2_/CF was placed under H_2_/Ar atmosphere at a heating rate of 2.5 °C min^−1^ to 410 °C and held for 1 h to obtain SnS/SnS_2_/SnO_2_/CF. SnS/SnS_2_/CF − 0.25–410 was prepared by placing SnS_2_/CF in a tube furnace by the same procedure. SnS/SnS_2_/CF, SnS/CF and SnS/SnS_2_/CF − 0.25–450, SnS/CF − 0.25–500 were prepared from SnS_2_/SnO_2_/CF and SnS_2_/CF at 450 and 500 °C, respectively, and the other conditions were the same as above.

### Preparation of Composite Coatings

In this work, Q235 mild steel was used as the substrate for the epoxy coating. Before use, the surface of Q235 steel is sandpapered smooth, then ultrasonically washed with ethanol, and finally dried. The SnS/SnS_2_/SnO_2_/CF composites were mixed with epoxy resin at 2 wt% loading, and n-butanol and xylene solvents were added and stirred evenly. Then, the polyimide curing agent was added and stirred for 0.5 h to make the sample and epoxy resin evenly mixed. Subsequently, the prepared coating solution was placed in a vacuum drying oven to remove bubbles. Finally, the composite coating solution was uniformly applied to the surface of the Q235 steel using a coating tool and placed in an oven to allow it to dry. Pure epoxy coatings were prepared without sample addition.

### Characterization

The crystal structure of the samples was described by X-ray diffraction (XRD, Rigaku Ultima IV, Cu Ka radiation, (*λ* = 0.51418)). The morphology, elemental distribution, and microstructure of the materials were observed by field emission scanning electron microscopy (F-SEM, JEOL JSM-7800F) transmission electron microscopy (TEM, JEOL JEM-2100) and high magnification transmission electron microscopy (HRTEM). Thermogravimetric analysis (TGA) was performed on an SDT Q600 analyzer with an air ramp rate of 10 °C min^−1^ from room temperature to 900 °C. Raman spectra are collected at 532 nm using a Renishaw InviaPlus micro-Raman spectroscopy system with a 50 mW DPSS laser. The surface elemental distribution of the material was characterized by X-ray photoelectron spectroscopy (XPS) on a Thermo Fisher ESCALAB 250Xi spectrometer using a 1486.6 eV Al Ka X-ray source. The EM parameters of each sample were determined using a vector network analyzer (Agilent N5234A, USA) using the coaxial method at test frequencies from 2 to 18 GHz, and the EMW absorption characteristics of the samples were further calculated. The sample was loaded 50 wt% in the oxygen resin, and n-butanol and xylene were added to the epoxy resin in the ratio of 3:7 and stirred well. Then an amount of polyimide curing agent was added and stirred well. Put the mixture into the preparation mold, and after the subsequent curing process, make a ring with an inner diameter of 3.04 mm and an outer diameter of 7 mm. The reflection loss of the material can be calculated by the following equations [41–44]:

1 $$ \begin{array}{*{20}c} {{\text{Z}}_{{{\text{in}}}} = Z_{0} \sqrt {\frac{{\mu_{r} }}{{\varepsilon_{r} }}} {\text{tan}}h\left| {j\left( {\frac{2\pi fd}{c}} \right)\sqrt {\varepsilon_{r} \mu_{r} } } \right|} \\ \end{array} $$

2 $$ \begin{array}{*{20}c} {{\text{RL}}\left( {{\text{d}}B} \right) = 20{\text{log}}\left| {\frac{{Z_{{{\text{in}}}} - Z_{0} }}{{Z_{{{\text{in}}}} + Z_{0} }}} \right|} \\ \end{array} $$

where *Z*_in_ and *Z*_0_ are the input impedance and free-space characteristic impedance of the standard wave-absorbing material, respectively, *f* is the frequency of the EMW, *d* is the thickness of the sample, and *c* is the velocity of the EMW in free space.

Through a typical three-electrode system, electrochemical corrosion experiments of samples and epoxy composite coatings in seawater solutions were measured on an electrochemical workstation CHI 760E. The Pt sheet was used as a counter electrode, Ag/AgCl as the reference electrode, and the coated electrode as the working electrode. The open circuit potential (OCP) behavior was recorded in the frequency range of 10^–2^ ~ 10^–5^ Hz and electrochemical impedance spectroscopy (EIS) measurements were performed. The seawater solution used was taken from the coast of Qingdao.

## Results and Discussion

**Fig. 1:**
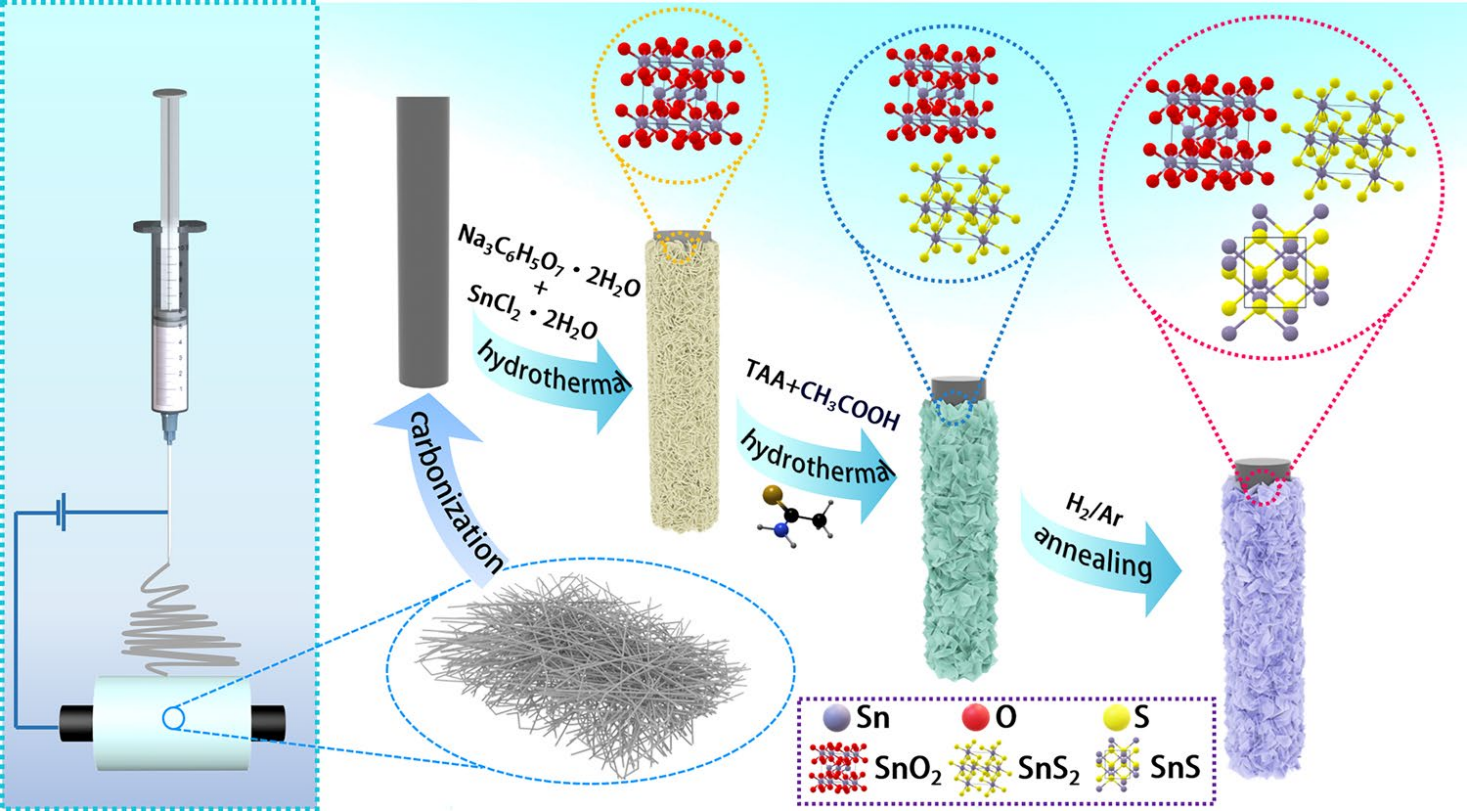
Schematic diagram of the synthesis process of SnS/SnS_2_/SnO_2_/CF

The preparation process of SnS/SnS_2_/SnO_2_/CF composites is illustrated in Fig. 1. Firstly, CF was obtained by electrostatic spinning and high-temperature calcination processes. Then, SnO_2_ nanosheets were grown on the surface of CF by hydrothermal method. In detail, SnCl_2_ dissolves to form Sn^2+^ and Cl^−^ ions, and trisodium citrate ionizes in solution to release citrate ions (C_6_H_5_O_7_^3−^) and sodium ions (Na^+^). Then, C_6_H_5_O_7_^3−^ complexes with Sn^2+^ ions to form a stable tin-citrate complex. Under high temperature and pressure, Sn^2+^ is oxidized to Sn^4+^. Sn^4+^ is further hydrolyzed and condensed to ultimately form SnO_2_, obtaining SnO_2_/CF. Subsequently, SnS_2_/SnO_2_/CF was prepared by a hydrothermal method by controlling the amount of TAA added. Specifically, SnO_2_/CF reacts in a mixed solution of acetic acid and water, and TAA releases H_2_S at high temperatures. CH_3_COOH reacts with SnO_2_ to dissociate Sn^4+^ with Sn^4+^ reacting with H_2_S to form SnS_2_ to obtain SnS_2_/SnO_2_/CF. Finally, under hydrogen/argon conditions, H_2_ acts as a reducing agent to effectively reduce SnS_2_ to SnS. Since the reduction temperature was low, SnS_2_ was partially reduced to SnS, and the coexistence of SnS_2_ and SnS was realized, thus obtaining the target product SnS/SnS_2_/SnO_2_/CF with a lamellar structure.

**Fig. 2:**
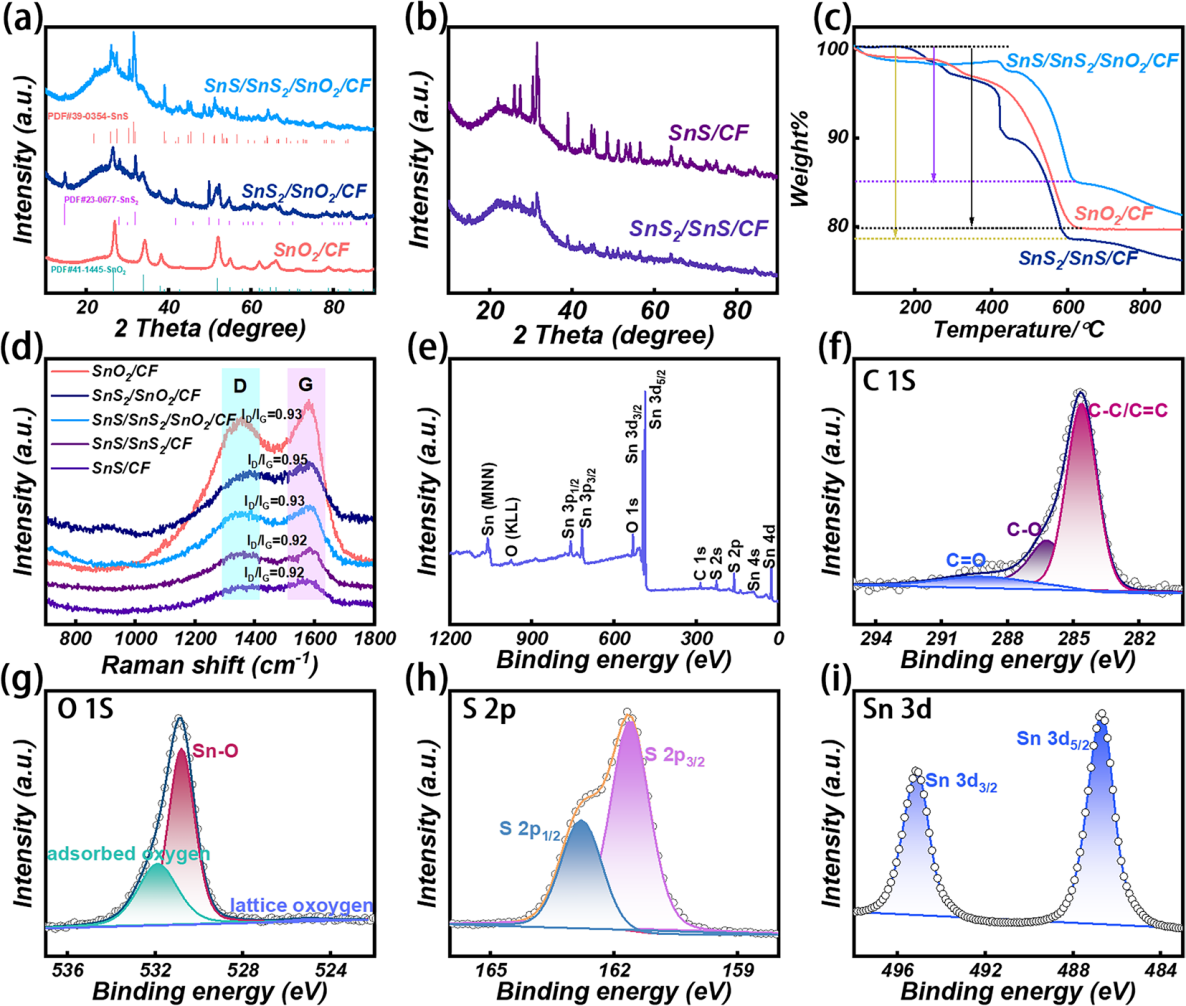
**a, b** XRD image of SnO_2_/CF, SnS_2_/SnO_2_/CF, SnS/SnS_2_/SnO_2_/CF, SnS/SnS_2_/CF, and SnS/CF. **c** Thermogravimetric images of SnO_2_/CF, SnS_2_/SnO_2_/CF, SnS/SnS_2_/SnO_2_/CF.** d** Raman image of SnO_2_/CF, SnS_2_/SnO_2_/CF, SnS/SnS_2_/SnO_2_/CF, SnS/SnS_2_/CF, and SnS/CF. **e-i** XPS spectra of SnS/SnS_2_/SnO_2_/CF sample

In Fig. 2a, the XRD images of SnO_2_/CF, SnS_2_/SnO_2_/CF, and SnS/SnS_2_/SnO_2_/CF are shown. The wide diffraction peaks at around 20°-30° correspond to amorphous carbon peaks. Diffraction peaks of SnO_2_ at 26.61°, 33.89°, 37.95°, 42.63°, 51.78°, 61.87°, 64.71°, and 78.71° correspond to its (110), (101), (200), (201), (211), (310), (112), and (310) crystal planes (PDF#41–1445), respectively. The diffraction peaks located at 15.05°, 28.19°, 32.12°, 41.88°, 49.96°, and 52.45° correspond to the (001), (100), (101), (102), (110), and (111) crystal planes of SnS_2_ (PDF#23–0677), respectively. The successful synthesis of SnS_2_/SnO_2_/CF composites was confirmed. The diffraction peaks of the (110), (120), (021), (101), (111), (040), (131), (002), and (211) crystal planes of SnS were located at 22.01°, 26.01°, 27.47°, 30.47°, 31.53°, 31.97°, 39.04°, 45.49°, and 48.51° (PDF#39–0354), respectively. Consequently, the above information confirms the successful synthesis of the multi-component tin compound SnS/SnS_2_/SnO_2_/CF composite. Figure 2b shows the XRD patterns of SnS/SnS_2_/CF and SnS/CF composites obtained at different pyrolysis temperatures of SnS_2_/SnO_2_/CF. The generation of SnS/SnS_2_/CF is due to the thermal decomposition of SnS_2_ at elevated temperatures to produce H_2_S and SnS. Subsequently, H_2_S further reacted with SnO_2_ to form SnS_2_ to obtain SnS/SnS_2_/CF composites. Further increasing the temperature, the H_2_ reducing ability is enhanced, and the conversion rate of SnS_2_ to SnS is significantly increased. At this point SnS_2_ can be completely reduced to SnS, giving SnS/CF. In this work, we also investigated the complete sulfidation of SnO_2_ to obtain SnS_2_/CF composites. The high-temperature thermal decomposition and reduction processes were then carried out at 410, 450, and 500 °C to obtain SnS/SnS_2_/CF − 0.25–410, SnS/SnS_2_/CF − 0.25–450, and SnS/CF − 0.25–500 (Fig. S1a). SnO_2_ is fully sulfidized and then reduced by thermal decomposition. At 410 and 450 °C this leads to partial reduction of SnS_2_ and at 500 °C SnS_2_ is completely reduced to SnS. The thermogravimetric analysis images of SnO_2_/CF, SnS_2_/SnO_2_/CF, and SnS/SnS_2_/SnO_2_/CF are shown in Fig. 2c. The mass loss of SnO_2_/CF is mainly due to the loss of carbon under air atmosphere. The loss of mass of SnS_2_/SnO_2_/CF and SnS/SnS_2_/SnO_2_/CF is due to the oxidation of SnS and SnS_2_ and the carbon combustion [45–48]. In the Raman spectrum (Fig. 2d), the D band (1350 cm^−1^) and G band (1590 cm^−1^) reflect the ordered/disordered carbon arrangement of the composite material. As can be seen in Figs. 2d and S1b, the graphitization degree of the samples is relatively high, which is mainly due to the influence of the CF skeleton. The crisscrossed CF network can enhance the effective transfer of electrons and has good conductivity loss, thereby promoting the absorption of EMW. The molecular structure and chemical state of the surface of the samples were analyzed by XPS. As shown in Fig. 2e, the characteristic peaks of Sn, O, C, and S can be observed in the range of 0–1200 eV. Sn (MNN) and O (KLL) represent the existence of Sn and O spiral peaks [49, 50]. C 1*s* at 284.62, 286.2, and 289.2 eV with three peaks representing C–C/C = C, C–O, and C = O, respectively (Fig. 2f) [51]. The diffraction peaks at positions 525.29, 530.96, and 531.99 eV in Fig. 2g are absorbed oxygen, Sn–O, and lattice oxygen, respectively [52]. S 2*p* has two diffraction peaks at 162.7 eV (S 2*p*_1/2_) and 161.5 eV (S 2*p*_3/2_) (Fig. 2h) [53]. Where based on previous reports [54, 55], the peak of S 2*p*_1/2_ corresponding to sulfur at the low coordination site is associated with S vacancies. The diffraction peaks of Sn 3*d* are shown in Fig. 2i, and the diffraction peaks at 486.5 and 494.9 eV correspond to Sn 3*d*_5/2_ and Sn 3*d*_3/2_, respectively [56]. The binding energy of S 2*p* in SnS/SnS_2_/SnO_2_/CF composites was decreased relative to that of pure SnS_2_ (161.69 and 162.85 eV). In addition, the Sn 3*d* binding energy in the SnS/SnS_2_/SnO_2_/CF composites was red-shifted compared to the pure SnS_2_ nanosheets (486.88 and 495.37 eV). All these evidences suggest the presence of sulfur vacancies [57–60]. The XPS spectra of SnO_2_/CF (Fig. S2a–d) displayed the elemental compositions (Sn, O, and C) of SnO_2_/CF. Among them, the content of O is higher, which confirms the successful synthesis of SnO_2_/CF. Figure S2e-i shows the XPS image of SnS_2_/SnO_2_/CF, where the presence of the elements Sn, O, C, and S can be seen in the total spectrum. The XPS spectra of SnS/SnS_2_/CF and SnS/CF are shown in Fig. S3, where the presence of elemental O is a result of unavoidable oxidation in air. The three diffraction peaks in the C 1*s* energy spectrum are attributed to C–C/C = C, C–O, and C = O. The diffraction peaks in the S 2*p* energy spectrum correspond to S 2*p*_1/2_ and S 2*p*_3/2_, and the diffraction peaks in Sn 3*d* correspond to Sn 3*d*_5/2_ and Sn 3*d*_3/2_.

**Fig. 3:**
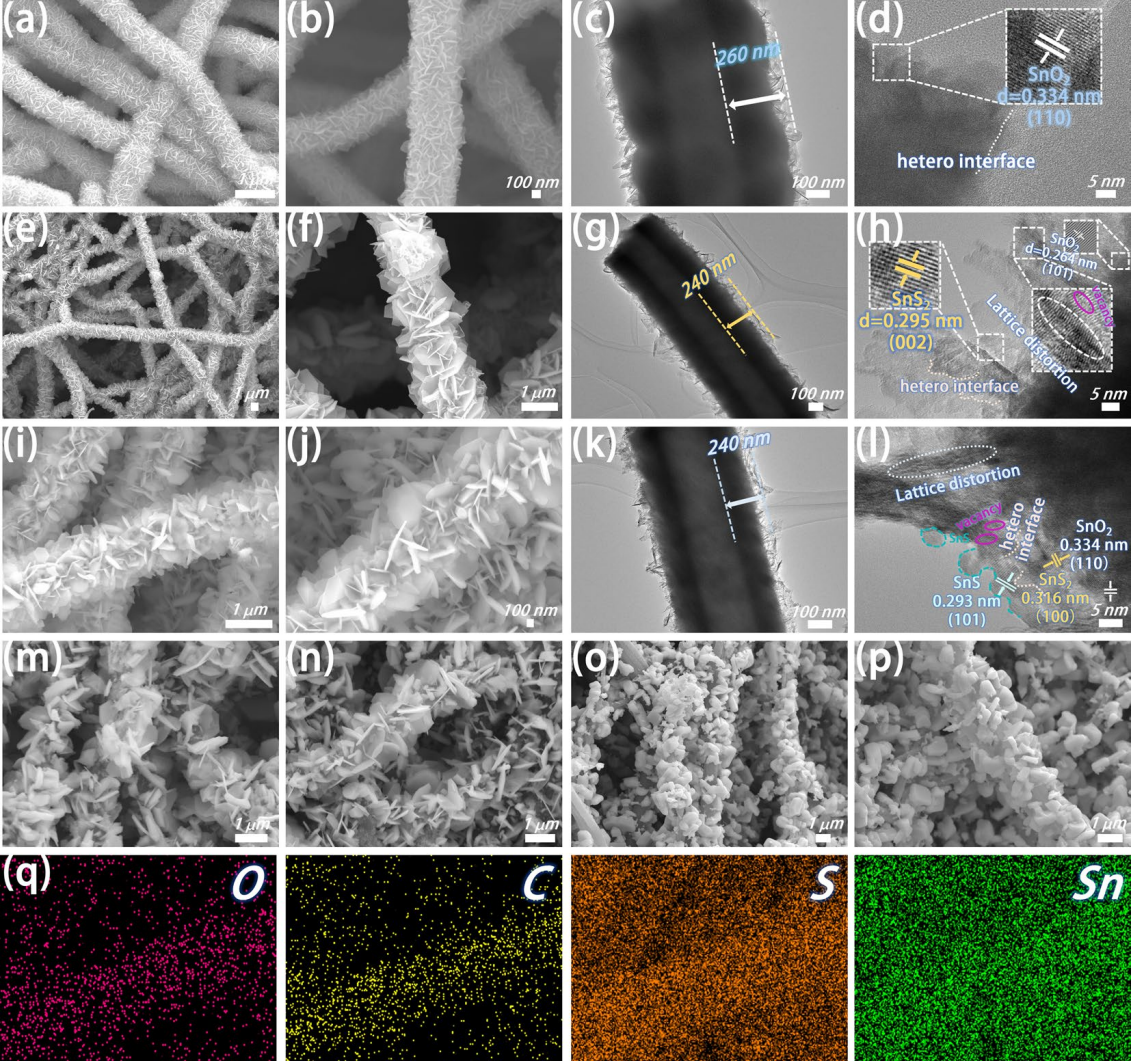
**a, b** SEM, **c** TEM, **d** HRTEM image of SnO_2_/CF. **e, f** SEM, **g** TEM, **h** HRTEM image of SnS_2_/SnO_2_/CF. **i, j** SEM, **k** TEM, **l** HRTEM image of SnS/SnS_2_/SnO_2_/CF. **m, n** SEM images of SnS/SnS_2_/CF. **o, p** SEM image of SnS/CF. **q** EDS mapping image of SnS/SnS_2_/SnO_2_/CF

Microscopic morphology and structure of the samples were observed by SEM and TEM. Figure S4a shows PANF with smooth surface and uniform size. Following pre-oxidation and high-temperature carbonization the obtained CF surface had some folds (Fig. S4b). SnO_2_ was closely wrapped around CF, forming SnO_2_/CF with a diameter of about 1 μm (Fig. 3a, b). The EDS image of SnO_2_/CF (Fig. S4c) shows the uniform distribution of Sn, O, and C elements along the fiber axis. As shown in Fig. 3c, SnO_2_ with a thickness of 260 nm is wrapped on the CF surface, and the morphology of the nanosheets can be seen more clearly at the edge. In the HRTEM image of SnO_2_/CF (Fig. 3d), the measured lattice spacing is 0.334 nm, corresponding to the (110) crystal plane of SnO_2_. After the sulfidation of SnO_2_/CF, part of SnO_2_ was converted into larger SnS_2_ nanosheets to obtain SnS_2_/SnO_2_/CF (Fig. 3e, f). In the TEM image of SnS_2_/SnO_2_/CF (Fig. 3g), SnS_2_/SnO_2_ is located in the outer layer of the fiber with a thickness of about 240 nm. In the HRTEM image of SnS_2_/SnO_2_/CF (Fig. 3h), the measured lattice spacings are 0.264 and 0.295 nm, indexed to the (101) crystal plane of SnO_2_ and the (002) crystal plane of SnS_2_, respectively. The existence of heterogeneous interfaces, lattice distortions, and discontinuous lattice fringes in SnS_2_/SnO_2_/CF can also be observed. The thickness of SnS/SnS_2_/SnO_2_/CF nanosheets obtained by thermal decomposition and reduction at 410 °C decreased slightly, but the overall change was not significant (Fig. 3i, j). The nanosheet structure can promote the scattering of EMW and extend the propagation path, thereby promoting the loss of EMW. Compared with the product of the previous step, the thickness of the fiber outer layer and the morphology of the nanosheets in SnS/SnS_2_/SnO_2_/CF remained basically unchanged (Fig. 3k). In the HRTEM image (Fig. 3l), SnS can be seen precipitating at the edge of the material. The measured lattice stripe spacings of 0.334, 0.316, and 0.293 nm point to the (110) crystal plane of SnO_2_, the (100) crystal plane of SnS_2_, and the (101) crystal plane of SnS, respectively. All information confirms the successful synthesis of SnS/SnS_2_/SnO_2_/CF. In SnS/SnS_2_/SnO_2_/CF materials, multiple materials contact to form heterogeneous interfaces with unique electronic properties and bonding states [61]. At the heterogeneous interfaces, non-homogeneous interfaces greatly impede the flow of charges [62]. The difference in dielectric properties leads to uneven charge distribution, resulting in an electric dipole moment, which enhances interfacial polarization and losses EMW. The amorphous structure results in the generation of discontinuous lattice streaks. Lattice distortion and discontinuous lattice fringes lead to the destruction of lattice symmetry in SnS/SnS_2_/SnO_2_/CF, resulting in lattice mismatches, and changes in electron distribution and charge transfer pathways. Furthermore, the presence of sulfur vacancies can also be inferred from the broken lattice at the marked circles. Sulfur vacancies can introduce defect energy levels that act as capture centers for electrons and holes, thereby changing the electronic and energy band structure of the material [63–65]. Thereby promoting interfacial polarization and EMW loss. The selected area electron diffraction (SAED) of SnO_2_/CF, SnS_2_/SnO_2_/CF, and SnS/SnS_2_/SnO_2_/CF are shown in Fig. S5. Figure 3m-p shows the SnS/SnS_2_/CF and SnS/CF composites obtained by thermal decomposition of SnS_2_/SnO_2_/CF at 450 and 500 °C. As the temperature increases, the nanosheets on the fiber surface begin to be destroyed. At 450 °C, the nanosheets on the surface of SnS/SnS_2_/CF material gradually fall off, but the basic nanosheet structure can be maintained. After thermal decomposition at 500 °C, the nanosheet structure on the fiber surface of SnS/CF material is destroyed. The nanosheets melt at high temperatures and form small stone-like structures. After SnO_2_ was thoroughly sulfurized, large SnS_2_ nanosheets tightly wrapped CF to form SnS_2_/CF composites (Fig. S6a). SnS_2_/CF contains Sn, S, and C elements distributed along the fiber (Fig. S6e, f). Subsequently, high-temperature thermal decomposition reduction was carried out at 410, 450, and 500 °C, to obtain SnS/SnS_2_/CF − 0.25–410 (Fig. S6b), SnS/SnS_2_/CF − 0.25–450 (Fig. S6c), and SnS/CF − 0.25–500 (Fig. S6d), respectively. The edges of the nanosheets begin to melt by heat at 410 °C. When the temperature rises to 450 °C the nanosheets wrapped around the fibers begin to fall off, and as the temperature continues to rise to 500 °C, the nanosheet structure of the fibers completely collapses. This demonstrated that the SnO_2_ component also works as an important role in structural stabilization.

**Fig. 4:**
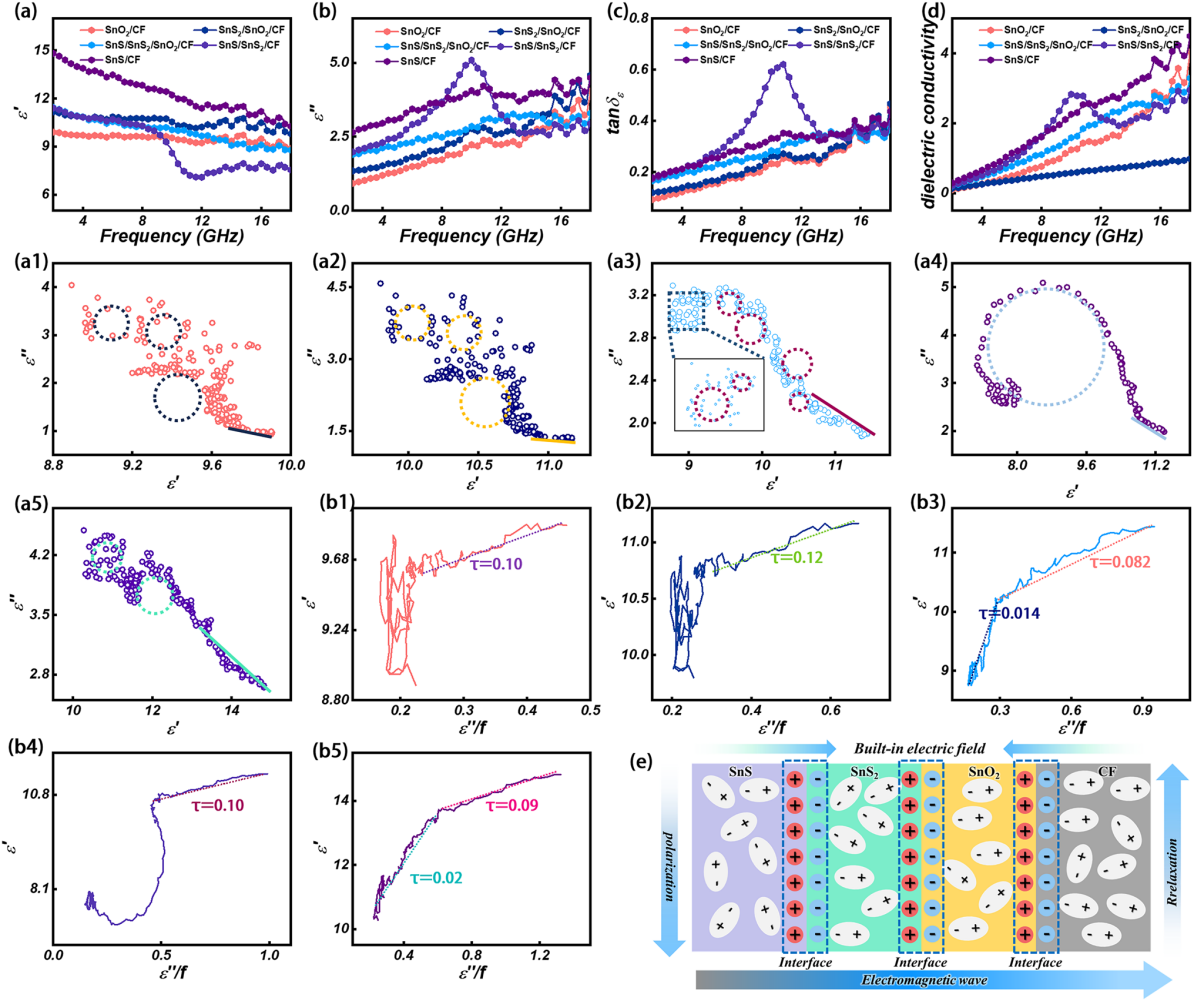
**a** *ɛʹ*, **b** *ɛ″*, **c** tan*δ*_*ɛ*_, **d** dielectric conductivity, **a1-a5** Cole–Cole, **b1-b5** *ɛʹ* versus *ɛ″/f* of SnO_2_/CF, SnS_2_/SnO_2_/CF, SnS/SnS_2_/SnO_2_/CF, SnS/SnS_2_/CF, and SnS/CF. **e** Interface polarization diagram of SnS/SnS_2_/SnO_2_/CF

Based on the EMW theory, the absorption performance of a wave-absorbing material is mainly intimately associated with its relative complex permittivity (*ε*_*r*_ = *ε'-jε"*) and magnetic permeability (*μ*_*r*_ = *μ′-jμ"*) [66–68]. The real part of the dielectric constant (*ε′*) reflects the energy storage capacity of the material, while the imaginary part (*ε″*) reveals its loss capacity [69, 70]. In this study, the magnetic storage and loss capacity of all samples can be neglected due to the lack of magnetic components. Therefore, the real (*µ'*) and imaginary (*µ″*) parts of permeability are taken as 1 and 0, respectively [71, 72]. For the fiber composites prepared in this work, the complex permittivity exhibits a frequency dependence [73]. As shown in Fig. 4a, the *ε′* values of all samples are in the range of 8.81 ~ 14.81 and show a downward trend in the test frequency range. In Fig. 4b, it is shown that the *ε*" values of all the samples show an increasing trend in the range of 2–18 GHz. Among them, the ε′ and ε″ values of SnS/CF are the largest. This is attributed to the unique layered structure, large interlayer spacing, high reversible capacity, and excellent conductivity of SnS. Especially note that the sample's dielectric curves with multiple polarization relaxation peaks can be observed in the ε' and ε'' plots This indicates the presence of a polarization relaxation process loss EMW. The dielectric loss tangent (tan*δ*_*ε*_) values of SnS/SnS_2_/CF and SnS/CF are larger, showing that they have stronger dielectric loss capability (Fig. 4c). The relevant electromagnetic parameters of SnS_2_/CF, SnS/SnS_2_/CF − 0.25–410, SnS/SnS_2_/CF − 0.25–450, and SnS/CF − 0.25–500 are shown in Fig. S7. Where SnS_2_/CF has the lowest dielectric parameter, indicating the worst dielectric loss capability. The dielectric parameters of the other samples do not differ much. The contribution of dielectric loss is mainly composed of conduction loss and polarization loss, while the polarization loss is mainly contributed by interface polarization and dipole polarization. The dielectric property related to the conduction loss can be calculated by Eq. (3) [74, 75]:

3 $$ \begin{array}{*{20}c} {\sigma = \omega \varepsilon_{0} \varepsilon^{\prime\prime}} \\ \end{array} $$

where ω (ω = 2π*f*) is the angular frequency and *ε*_*0*_ (*ε*_*0*_ = 8.854 × 10^–12^ F m^−1^) is the free-space dielectric constant. The conductivity of SnS_2_/SnO_2_/CF is relatively small, and other samples have multiple peaks at high frequencies (Fig. 4d). This is due to the influence of polarization relaxation. The polarization relaxation process is further investigated by Deby’s theory and combined with Eqs. (4) and (5) to study the Cole–Cole semicircle [76, 77]:

4 $$ \begin{array}{*{20}c} {\varepsilon^{\prime} = \varepsilon_{\infty } + \frac{{\varepsilon_{s} - \varepsilon_{\infty } }}{{1 + \left( {2\pi f} \right)^{2} r^{2} }}} \\ \end{array} $$

5 $$ \begin{array}{*{20}c} {\varepsilon^{\prime\prime} = \frac{{\omega \tau \left( {\varepsilon_{s} - \varepsilon_{\infty } } \right)}}{{1 + \left( {2\pi f} \right)^{2} r^{2} }}} \\ \end{array} $$

where *ε*_*s*_ is the static dielectric constant and *ε*_*∞*_ is the relative dielectric constant at infinite frequency. The long smooth straight line on the *ε′*-*ε″* image curve is related to the conduction loss (tail solid line), while the semicircle represents the polarization relaxation process (dotted circle) [78]. SnS/SnS_2_/SnO_2_/CF has more semicircles compared to the other materials (Fig. 4a1-a5), which indicates that it has more polarization relaxation processes. This is attributed to the fact that it has more component materials and more heterogeneous interfaces. Due to the differences in work functions of different components in the composite system, carriers tend to migrate from the phase with higher work function to the phase with lower surface work function. The surface electron affinity of SnO_2_ oxide is high, and the electron escapes from the surface requires high energy, and its work function is high. SnS_2_ increases the negative charge density because of the presence of sulfur atoms on the surface, resulting in a higher work function. The electronic structure of SnS is closer to the metallic state, so the work function is lower [79]. Carrier migration between different components is accompanied by EM energy consumption. The electron transfer at the interface between the two materials leads to the formation of a built-in electric field and a space charge region, which affects charge transport and interface polarization [80]. Opposite charges accumulate at the heterogeneous interface, forming a built-in electric field that promotes electron transport and dissipates EMW through interfacial polarization effects (Fig. 4e). Meanwhile, the presence of S vacancies in SnS/SnS_2_/SnO_2_/CF can act as the active site of the dipole. The center of symmetry is deflected in the presence of an applied electromagnetic field, producing a strong dipole polarization. We further study the polarization relaxation process by Eq. (6) [81–83]:

6 $$ \begin{array}{*{20}c} {\varepsilon^{\prime} = \frac{1}{2\pi \tau }\frac{{\varepsilon^{\prime\prime}}}{f} + \varepsilon_{\infty } } \\ \end{array} $$

Followed by the relationship between *ε′* and *ε″*/*f* can be concluded the time *τ* of polarization relaxation. As shown in Fig. 4b1-b5, the relaxation times of SnO_2_/CF, SnS_2_/SnO_2_/CF, SnS/SnS_2_/SnO_2_/CF, SnS/SnS_2_/CF, SnS/CF were 0.10, 0.12, (0.014 and 0.082), 0.10, (0.02 and 0.09), respectively. It is worth noting that different materials have different polarization relaxation times. Among them, SnS/SnS_2_/SnO_2_/CF and SnS/CF composites have multiple polarization relaxation times, which indicates the existence of multiple polarization relaxation processes [84]. This is attributed to the fact that each polarization relaxation process possesses a unique relaxation time and exhibits multiple EMW loss characteristics. Defects and other heteroatoms in the material act as polarization sites capable of inducing dipole polarization phenomena in the presence of an applied magnetic field. Simultaneously, the interfacial polarization is induced due to the interaction of SnS, SnS_2_, SnO_2_, and CF with each other at the interface, which further enhances the EMW absorption ability of the material. The attenuation constant *α* is used to characterize the attenuation properties of the material to EMW and is calculated using Eq. (7) [85–87]:

7 $$ \begin{array}{*{20}c} {\alpha = \frac{\sqrt 2 \pi f}{c} \times \sqrt {\left( {\mu^{\prime\prime}\varepsilon^{\prime\prime} - \mu^{\prime}\varepsilon{\prime} } \right) + \sqrt {\left( {\mu^{\prime}\varepsilon^{\prime\prime} + \mu^{\prime\prime}\varepsilon^{\prime}} \right)^{2} + \left( {\mu^{\prime\prime}\varepsilon^{\prime\prime} - \mu^{\prime}\varepsilon^{\prime}} \right)^{2} } } } \\ \end{array} $$

As shown in Fig. S8a, the change in the *α* value of the material is consistent with the changing trend of the dielectric constant, which proves that dielectric loss is the main mechanism of EMW attenuation. SnS/SnS_2_/CF and SnS/CF have large *α* values, indicating that they have strong EMW attenuation ability. However, EMW absorption performance is determined by several factors, especially impedance matching. The |*Z*_in_/*Z*_0_| values of SnS/SnS_2_/SnO_2_/CF and SnS/SnS_2_/CF are closest to 1, which may have excellent impedance matching performance (Fig. 8b).

**Fig. 5:**
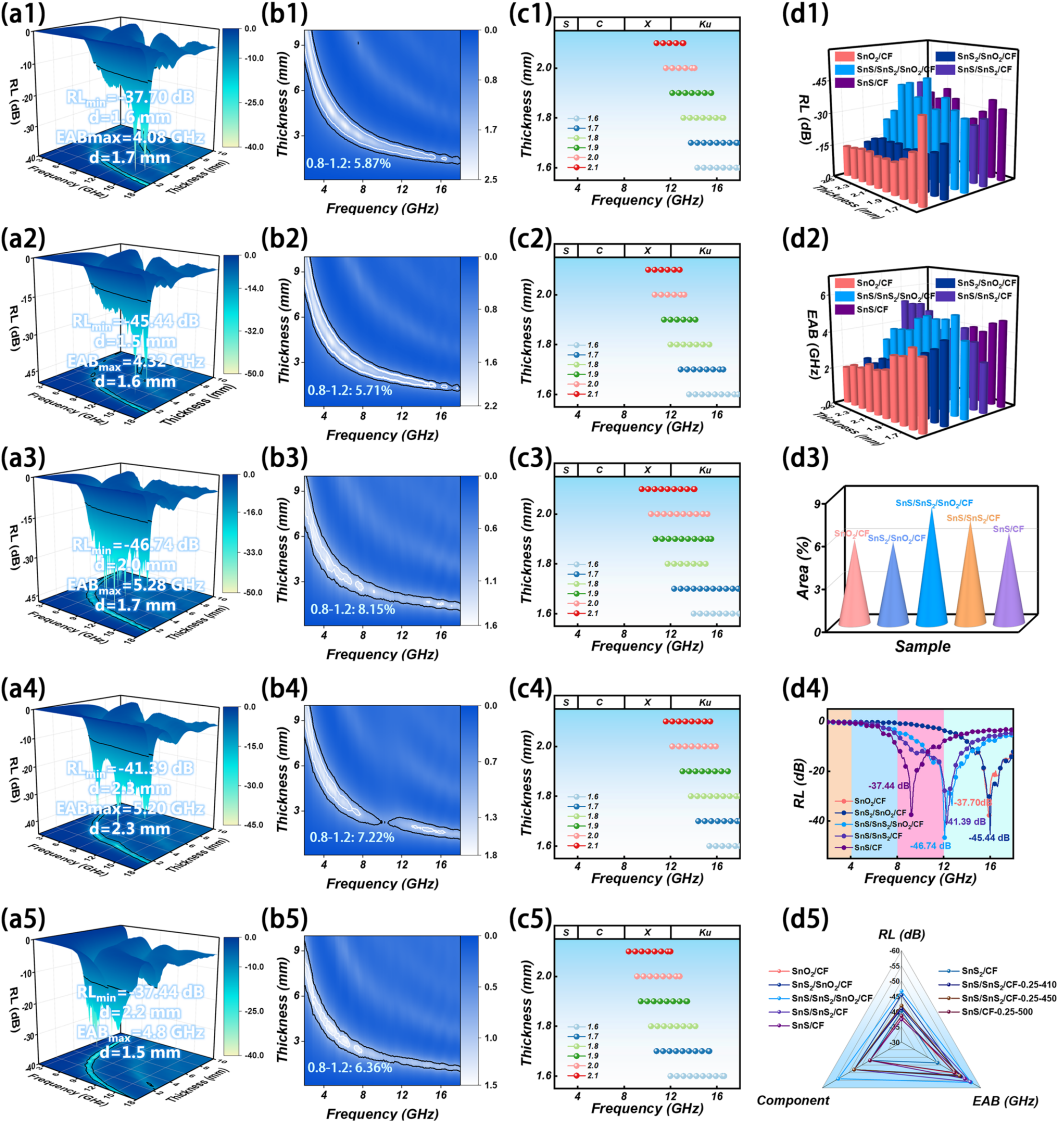
EMW absorption performance of SnO_2_/CF, SnS_2_/SnO_2_/CF, SnS/SnS_2_/SnO_2_/CF, SnS/SnS_2_/CF, SnS/CF. **a1-a5** 3D RL, **b1-b5** 2D impedance matching, **c1-c5** impedance matching at different thicknesses, **d1** 3D RL and **d2** EAB at 1.6 to 2.5 mm, **d3** 3D impedance matching and **d4** 2D RL at matching thickness. **d5** Radar map of RL, EAB, and component relationships for all samples

As shown in Figs. 5a1-a5 and S9, S10, SnS/CF has the best EMW absorption performance (RL_min_ = 37.44 dB, EAB_max_ = 4.80 GHz) in the binary system material. Among the ternary system materials, SnS/SnS_2_/CF has the most excellent EMW absorption performance, with an EAB_max_ of 5.20 GHz, while SnS_2_/SnO_2_/CF has an EAB_max_ of only 4.32 GHz. This is because SnS_2_ generates S vacancies during its reduction to SnS, which promotes the generation of polarization relaxation. Nevertheless, the EMW absorption performance of SnS_2_/SnO_2_/CF is still better than that of SnS/CF. This suggests that the EMW absorption performance can be enhanced by constructing multiple heterointerfaces by increasing the components. The SnS/SnS_2_/SnO_2_/CF composites are obtained by further increasing the components. The EMW absorption performance is further enhanced due to the increased heterogeneous interface and S vacancies effect, with an RL_min_ of − 46.74 dB at a matched thickness of 2.0 mm and an EAB_max_ of 5.28 GHz at 1.7 mm. The results show that the EMW attenuation performance can be effectively improved by adjusting the multiple loss mechanism in the composite system to construct multiple heterogeneous interfaces. The EMW absorption performance of a material has an important relationship with impedance matching, which indicates the ability of EMW to enter the material without being reflected [88, 89]. *Z*_in_*/Z*_0_ in the region of 0.8–1.2 is the ideal impedance matching performance. Figure 5b1-b5 shows the area occupied by SnO_2_/CF, SnS_2_/SnO_2_/CF, SnS/SnS_2_/SnO_2_/CF, SnS/SnS_2_/CF, and SnS/CF impedance matches in the range of 0.8–1.2, are 5.87%, 5.71%, 8.15%, 7.22%, and 6.36%, respectively. Combined with Fig. 5d3 it can be seen that SnS/SnS_2_/SnO_2_/CF has the largest area, which indicates the optimum impedance matching. Its superior impedance matching performance comes from its own characteristics and non-uniform interface structure. The EAB at various thicknesses are shown in Fig. 5c1-c5, and it can be seen that at thin matching thicknesses, the EAB of the material is mainly concentrated in the X and Ku bands. The relationship between the matching thickness of the material and the reflection loss and frequency is studied by the quarter-wavelength theory, the equation is as follows [90–93]:

8 $$ \begin{array}{*{20}c} {t_{m} = \frac{n\lambda }{4} = \frac{nc}{{4f_{m} \sqrt {\left| {\mu_{r} } \right|\left| {\varepsilon_{r} } \right|} }}\left( {n = 1,3,5 \ldots } \right)} \\ \end{array} $$

where *λ* is the wavelength of EMW, *c* is the propagation speed of EMW in vacuum, and |*μ*_r_| and |*ε*_*r*_| are the moduli of *μ*_*r*_ and *ε*_*r*_, respectively. The matched thickness of RL moves in a decreasing direction with increasing frequency and falls exactly on the *λ*/4 curve (Fig. S11). This suggests that RL corresponds to a matching thickness that corresponds perfectly to the quarter-wavelength theory. The SnS/SnS_2_/SnO_2_/CF composite exhibits the smallest RL and the largest EAB at the thin thickness (Fig. 5d1, d2), which further confirms its excellent EMW absorption properties. The SnS/SnS_2_/SnO_2_/CF sample has the strongest RL, covering part of the X-band and Ku-band (Fig. 5d4). By comparing the relationships among RL, EAB, and components of all samples in this work, it was found that SnS/SnS_2_/SnO_2_/CF samples with the most components had the best EMW absorption performance (Fig. 5d5). Comparing the SnS/SnS_2_/SnO_2_/CF material prepared in this study with other CF-based and Sn-based materials, it can be seen that it has a small RL and a large EAB. It has significant advantages in EMW absorption (Fig. S12, Table S1). Therefore, it is an effective strategy to improve the absorption performance of EMW by rationally adjusting the microstructure and chemical components.

**Fig. 6:**
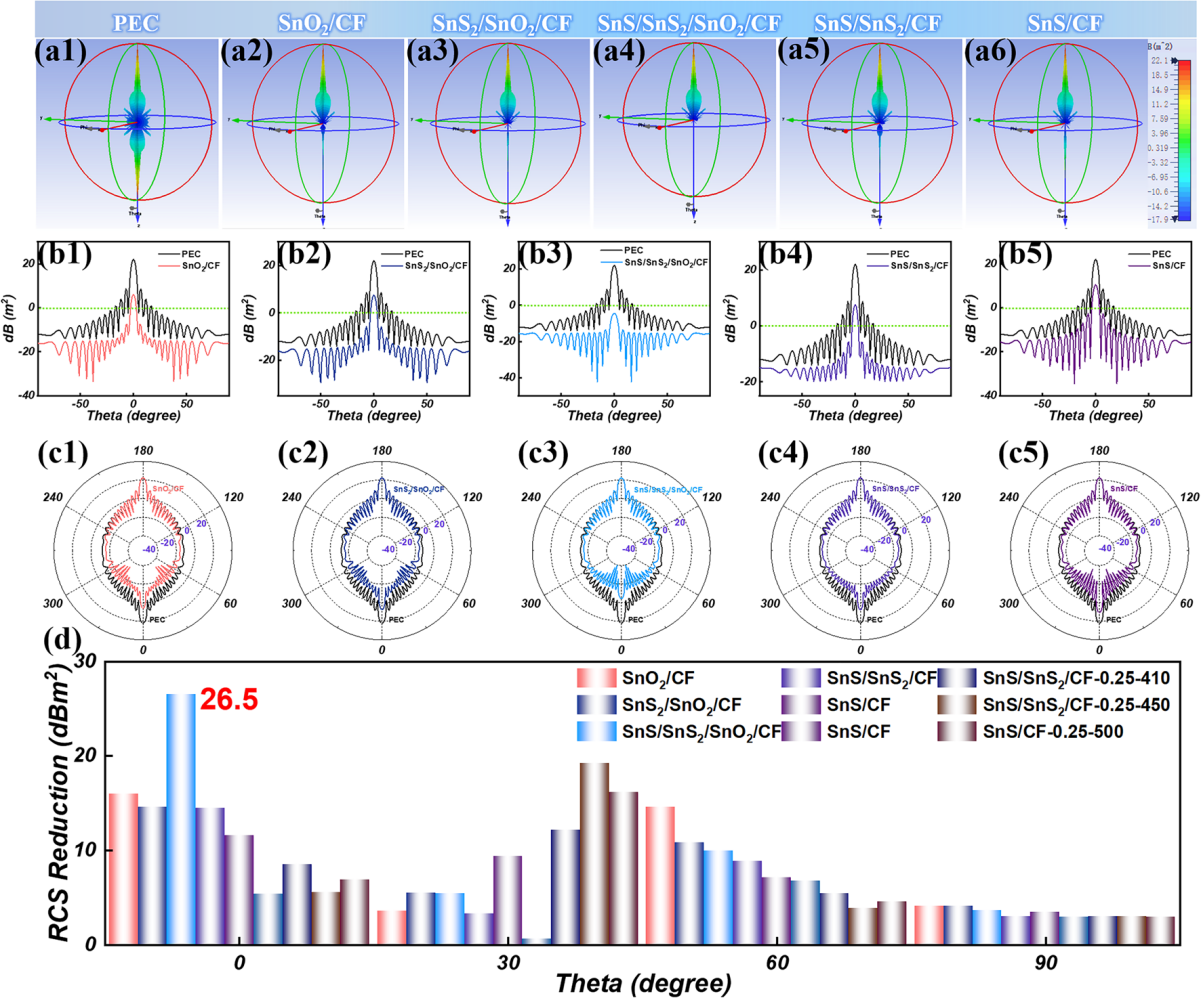
**a1–a6** 3D radar wave scattering signals, **b1–b5** RCS simulation curves, and **c1–c5** RCS simulation results of polar coordinates of PEC, SnO_2_/CF, SnS_2_/SnO_2_/CF, SnS/SnS_2_/SnO_2_/CF, SnS/SnS_2_/CF, SnS/CF. **d** RCS reduction value of the all samples

The radar cross section (RCS) of the composites was simulated by CST Studio Suite software at 12.16 GHz to explore the application prospect of the materials in the real environment [94–97]. The 3D radar wave scattering signals of the perfect electric conductor (PEC) and all samples are shown in Figs. 6a and S13a. It can be clearly seen that the radar scattering signal intensity of the composite material is reduced compared with that of PEC. It is particularly noteworthy that the radar scattering signal intensity of the SnS/SnS_2_/SnO_2_/CF sample is the lowest, indicating that it has a strong EMW attenuation ability. Figures 6b, c and S13b, c show the RCS values of pure PEC and all samples in different angle ranges. In the range of − 90° ~ 90°, the RCS values of all the samples were reduced to different degrees relative to the pure PEC. The RCS results show that the SnS/SnS_2_/SnO_2_/CF sample have good attenuation ability for EMW and can effectively suppress the radar scattering signals. The EMW attenuation capability of the prepared samples was further investigated by calculating the RCS reduction values at different detection angles (0°, 30°, 60°, 90°) (Fig. 6d). The maximum value of reduction in RCS value for SnS/SnS_2_/SnO_2_/CF sample is 26.5 dB m^2^ at 0°. This further confirms that the SnS/SnS_2_/SnO_2_/CF sample has strong EMW attenuation capability and has great potential in real-world situations such as radar stealth [98–100].

**Fig. 7:**
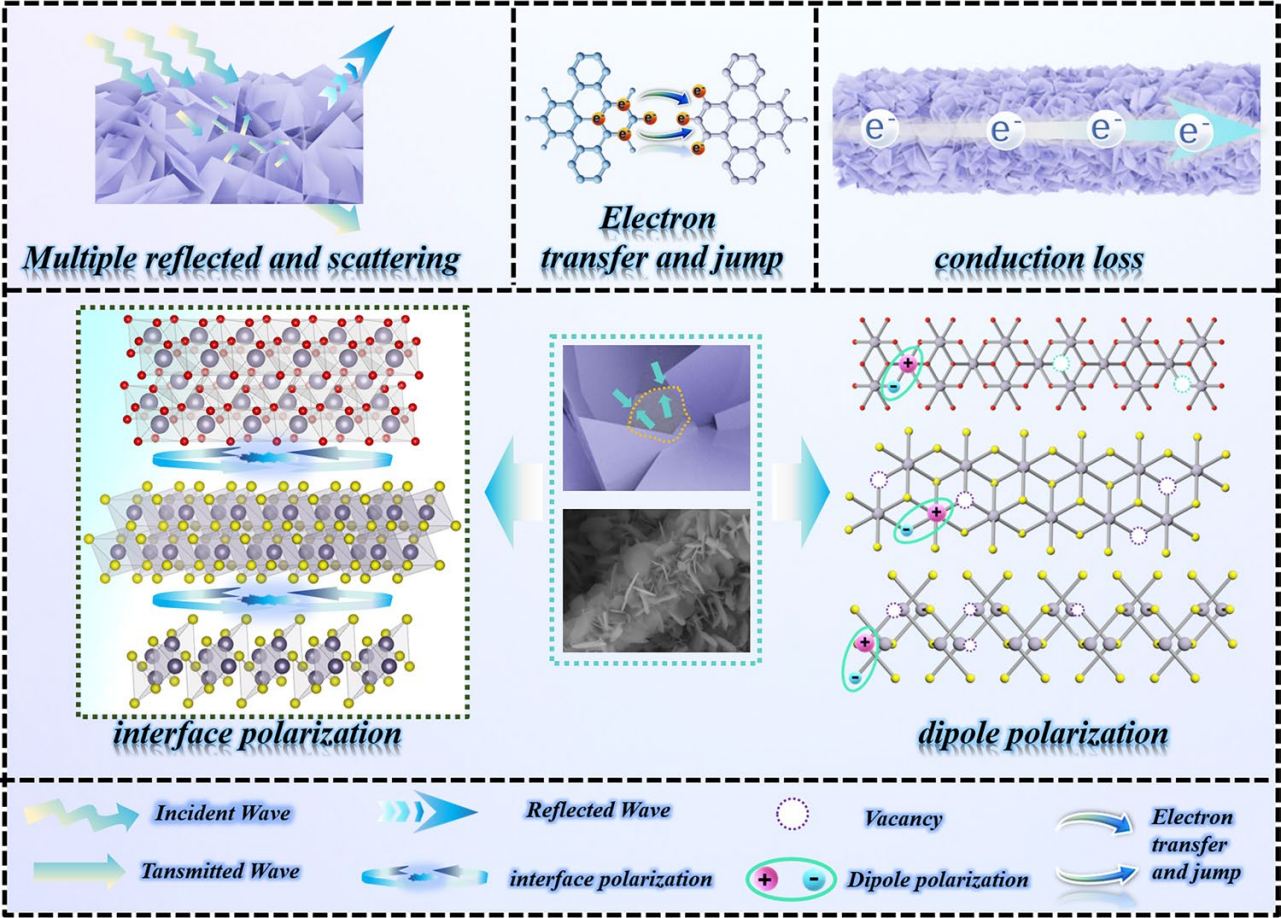
Electromagnetic wave absorption mechanism

Figure 7 shows the mechanism of the EMW absorption of SnS/SnS_2_/SnO_2_/CF composites. Firstly, the nanosheet structure can effectively scatter EMW, which effectively prolongs the propagation path of EMW and promotes the absorption of EMW. Secondly, the CF skeletons are composite with tin compounds with electrical conductivity to form an efficient conduction network. As EMW propagates through the conduction network, electrons in the material are stimulated, prompting them to migrate and jump, which in turn creates an electric current. This process dissipates EMW energy in the form of heat, which significantly improves the conduction loss capability of the material [101]. The introduction of tin compounds changes the dielectric parameters of the sample and optimizes the impedance matching. Furthermore, the presence of multiple non-uniform interfaces promotes the accumulation of positive and negative charges at the interfaces, creating a model that resembles an equivalent capacitor structure. The charges at the interface cannot be fully synchronized to respond to the alternating frequency of the EMW under the action of the electromagnetic field. This results in a polarization relaxation process, which in turn enhances the dielectric loss capability of the material to EMW. The material also has vacancies and lattice defects capable of producing dipoles. Under the induction of an electromagnetic field, the dipole moments are rearranged with the direction of the electric field, and the sum of the dipole moment vectors is not zero, giving rise to the dipole polarization effect [102–105]. In summary, SnS/SnS_2_/SnO_2_/CF composites absorb EMW through the synergistic action of multiple loss mechanisms.

**Fig. 8:**
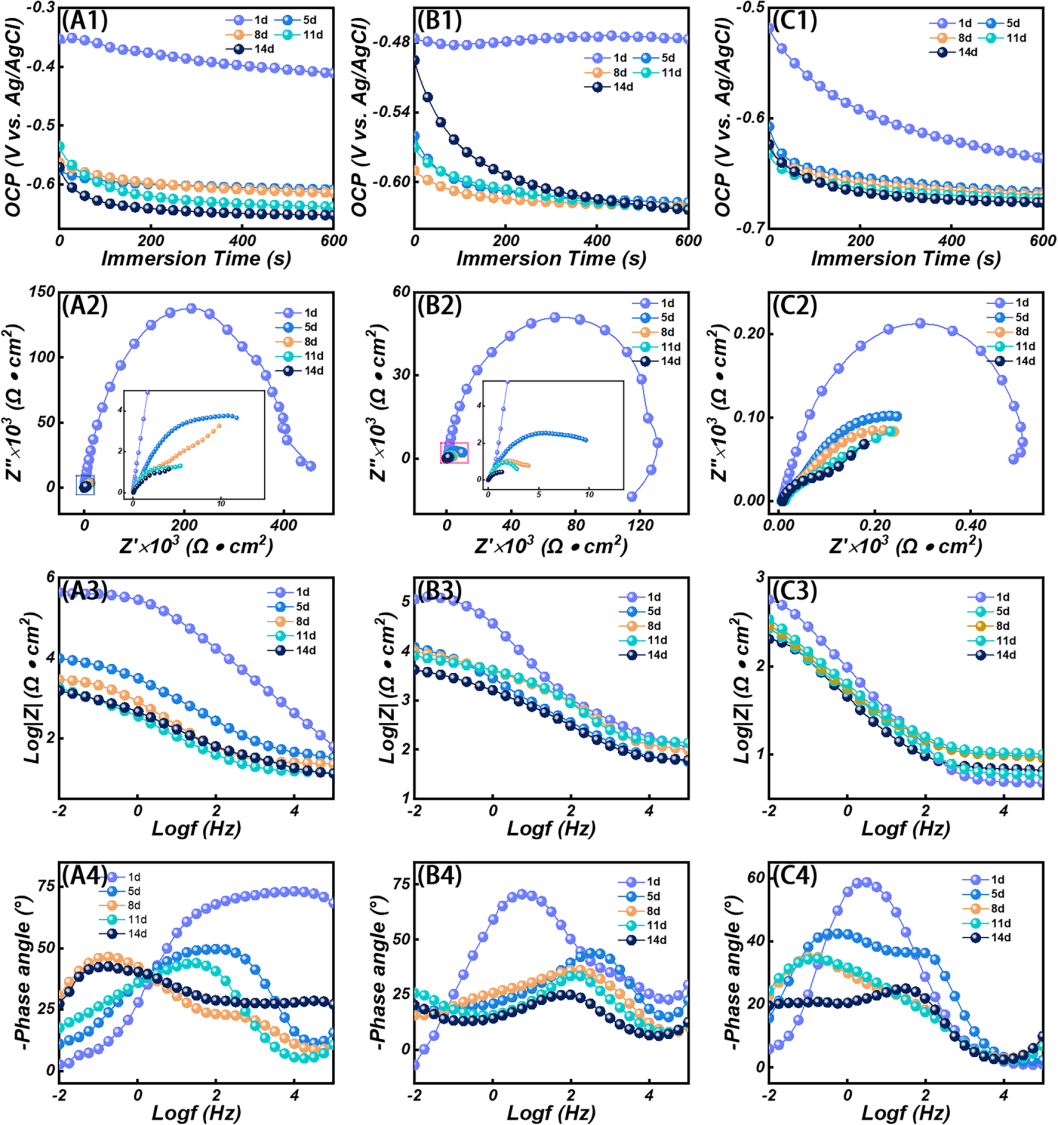
**a1–c1** OCP values, **a2–c2** Nyquist plots, **a3–c3** Bode plots, **a4–c4** Bode plots of SnS/SnS_2_/SnO_2_/CF composite coating, pure epoxy coating, and Q235 steel

For applications in complex environments, especially in the marine environment, this places higher demands on EMW absorbers. The coating’s corrosion protection is assessed by a chemical measurement technique using a three-electrode system. Firstly, the variation of open circuit potential (OCP) of the material with immersion time was investigated. Generally, higher OCP values have a lower tendency to corrode [106–110]. The composite coating with SnS/SnS_2_/SnO_2_/CF samples has the highest OCP value, and the bare steel coating has the lowest OCP value (Fig. 8a1-c1). The results show that the corrosion resistance of SnS/SnS_2_/SnO_2_/CF composite coating is the best, followed by pure epoxy coating, and bare steel coating is the worst. With the extension of immersion time, after two weeks of immersion, the corrosive medium gradually penetrated the coating through the coating defects. This results in a reduction in the corrosion resistance of the coating, along with a corresponding reduction in the OCP value. Figure 8a2-c2 shows the electrochemical impedance spectroscopy (EIS) of SnS/SnS_2_/SnO_2_/CF and epoxy resin composite coating, pure epoxy resin, and bare steel, respectively. From the plots, it can be shown that the capacitive arc radii of all three coatings exhibit larger values on the first day. Noteworthy, the SnS/SnS_2_/SnO_2_/CF composite coating reached a capacitive arc maximum of 1.40 × 10^5^, which proves their strong corrosion resistance. The bare steel has the smallest capacitive arc radius, indicating the worst corrosion resistance. With time the radius of the capacitive arc decreases, which shows a gradual decrease in the corrosion protection of the coating. However, the coating containing SnS/SnS_2_/SnO_2_/CF always maintained the largest capacitive arc radius throughout the process, which fully demonstrated its excellent corrosion resistance. The radius of the capacitor arc decreases sharply from day 5 and changes little in the following days. This indicates that the coating is beginning to be damaged and that the corrosion of the coating becomes more severe with increasing immersion time. This is mainly due to the penetration of oxygen, water, and corrosive ions (Cl^−^) into the coating, which ultimately results in the corrosion process occurring at the junction of the coating and the Q235 steel substrate. Normally, the impedance modulus at the lowest frequency of the coating (|*Z*|_0.01 Hz_) is used as a semi-quantitative indicator to assess the corrosion resistance of the coating. The impedance modulus in the Bode plot reflected the ability of the coating to suppress the current between the cathode and anode. The larger the impedance modulus of the coating, the more effective the corrosion protection [111, 112]. The |*Z*|_0.01Hz_ values of SnS/SnS_2_/SnO_2_/CF composite coating, pure epoxy, and bare steel are presented in Fig. 8a3-c3. On the first day, their impedance modulus at low frequencies was 1 × 10^5.65^, 1 × 10^5.08^, and 1 × 10^2.74^ Ω cm^2^, respectively. Among them, the impedance modulus of the SnS/SnS_2_/SnO_2_/CF composite coating was the largest, which indicated that they had the strongest ability to block the penetration of corrosive substances. The impedance modulus decreases with increasing immersion time. After soaking for 14 days, the impedance modulus of the three coatings decreased to 1 × 10^3.19^, 1 × 10^3.61^, and 1 × 10^2.29^ Ω cm^2^, respectively. This phenomenon indicates that the corrosion resistance of the coating decreases. The change of coating properties was studied by studying the phase Angle of each coating, and the phase Angle of the coating decreased with the increase of soaking time (Fig. 8a4-c4). There is also a gradual shift of the time constant toward the low-frequency region, both of which indicate that corrosion of the coating has occurred [113–116].

**Fig. 9:**
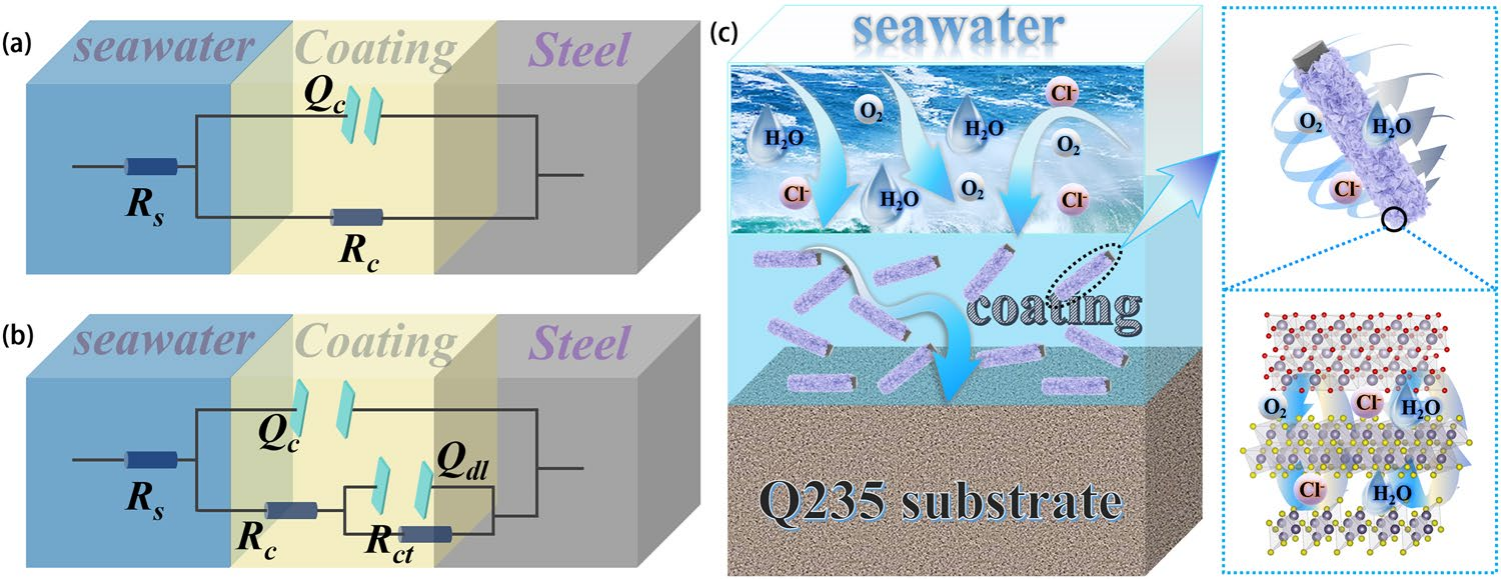
**a, b** Equivalent circuit models of coatings at different stages. **c** Corrosion prevention mechanism diagram of SnS/SnS_2_/SnO_2_/CF composite coating

The corresponding equivalent circuit model when the corrosion medium before reaching the metal substrate is shown in Fig. 9a. On the contrary, when the corrosive medium reaches the metal matrix, it reacts with the metal and charge transfer occurs, resulting in corrosion of the Q235 steel substrate (Fig. 9b). Figure S14 shows SEM images of SnS/SnS_2_/SnO_2_/CF composite coating and pure epoxy coating after 14 days of immersion. Following prolonged immersion, the SnS/SnS_2_/SnO_2_/CF composite coating was able to maintain intact coating morphology with and without significant damage. The pure epoxy resin coating has been damaged, and a large number of bubbles are produced on the surface, indicating that the coating has been severely corroded. The above information indicates that the addition of SnS/SnS_2_/SnO_2_/CF composites to epoxy resin is beneficial to improve the corrosion resistance of the coating. Figure 9c shows the anti-corrosion mechanism of SnS/SnS_2_/SnO_2_/CF composite coating. Adding SnS/SnS_2_/SnO_2_/CF to epoxy resin coating can improve the density of the coating. It can effectively prevent water molecules, oxygen, Cl^−^ and other corrosive media from passing through the coating. The addition of fiber materials can improve the mechanical strength of the coating, thus increasing the coating’s service life and protective properties. Carbon fiber composites also have a certain degree of electrical conductivity, achieving cathodic protection and reducing the corrosion rate of the substrate. Thereby achieving anti-corrosion properties [117–121].

## Conclusions

In summary, SnS/SnS_2_/SnO_2_/CF one-dimensional (1D) composites with multi-heterogeneous interfaces were ingeniously constructed by component modulation engineering. The 1D structure establishes the conduction network and promotes the conduction loss. The multi-component materials form multiple heterogeneous interfaces to enhance polarization relaxation and effectively regulate impedance matching, thereby achieving excellent EMW absorption performance. The SnS/SnS_2_/SnO_2_/CF composite material achieves strong absorption with an RL_min_ of − 46.74 dB at a thickness of 2.0 mm. The EAB_max_ at 1.7 mm is 5.28 GHz, realizing a wide bandwidth. At the same time, the coatings with added SnS/SnS_2_/SnO_2_/CF composites were shown by electrochemical tests to have excellent corrosion resistance, which hindered the corrosion reaction by delaying the penetration of corrosive media. In this work, high-efficiency EMW absorption and good corrosion protection were achieved by modulating the material components. This provides a new direction for the development of EMW absorbers with excellent EMW absorption properties and corrosion resistance.

## Supplementary Information

Below is the link to the electronic supplementary material.

Supplementary file1 (DOCX 7199 kb)

## References

[1] X.G. Su, J. Wang, T. Liu, Y.N. Liu, B. Zhang et al., Controllable atomic migration in microstructures and defects for electromagnetic wave absorption enhancement. Adv. Funct. Mater. **34**, 2403397 (2024). 10.1002/adfm.202403397

[2] X. Zhong, M.K. He, C.Y. Zhang, Y.Q. Guo, J.W. Hu et al., Heterostructured BN@Co-C@C endowing polyester composites excellent thermal conductivity and microwave absorption at C band. Adv. Funct. Mater. **34**, 2313544 (2024). 10.1002/adfm.202313544

[3] T.B. Ma, Y.L. Zhang, K.P. Ruan, H. Guo, M.K. He et al., Advances in 3D printing for polymer composites: a review. InfoMat **6**, e12568 (2024). 10.1002/inf2.12568

[4] A.L. Feng, X. Zhu, Y.N. Chen, P.T. Liu, F.B. Han et al., Functional biomass-derived materials for the development of sustainable batteries. ChemElectroChem **11**(13), e202400086 (2024). 10.1002/celc.202400086

[5] K.J. Gong, Y.M. Peng, A. Liu, S.H. Qi, H. Qiu, Ultrathin carbon layer coated MXene/PBO nanofiber films for high performance electromagenetic interference shielding and thermal stability. Compos. Part A **176**, 107857 (2024). 10.1016/j.compositesa.2023.107857

[6] J.L. Liu, L.M. Zhang, H.J. Wu, Anion-doping-induced vacancy engineering of cobalt sulfoselenide for boosting electromagnetic wave absorption. Adv. Funct. Mater. **32**(26), 2200544 (2022). 10.1002/adfm.202200544

[7] X.G. Su, Y. Zhang, J. Wang, Y.Q. Liu, Enhanced electromagnetic wave absorption and mechanical performances of graphite nanosheet/PVDF foams via ice dissolution and normal pressure drying. J. Mater. Chem. C **12**, 7775–7783 (2024). 10.1039/d4tc00929k

[8] N. Qu, G.X. Xu, Y.K. Liu, M.K. He, R.Z. Xing et al., Multi-scale design of metal-organic framework metamaterials for broad-band microwave absorption. Adv. Funct. Mater. **34**, 2402923 (2024). 10.1002/adfm.202402923

[9] Z.Z. He, H.X. Xu, L.Z. Shi, X.R. Ren, J. Kong et al., Hierarchical Co_2_P/CoS_2_@C@MoS_2_ composites with hollow cavity and multiple phases toward wideband electromagnetic wave absorption. Small **20**(6), 2306253 (2024). 10.1002/smll.202306253 10.1002/smll.202306253 37771205

[10] X.D. Li, X. Zhu, A.L. Feng, M.M. An, P.T. Liu et al., Electrochemical and surface analysis investigation of corrosion inhibition performance: 6-Thioguanine, benzotriazole, and phosphate salt on simulated patinas of bronze relics. J. Mater. Res. Technol. **29**, 5667–5680 (2024). 10.1016/j.jmrt.2024.03.001

[11] X. Yang, L.X. Xuan, W.W. Men, X. Wu, D. Lan et al., Carbonyl iron/glass fiber cloth composites: achieving multi-spectrum stealth in a wide temperature range. Chem. Eng. J. **491**, 151862 (2024). 10.1016/j.cej.2024.151862

[12] C. Wang, Y. Liu, Z. Jia, W. Zhao, G. Wu, Multicomponent nanoparticles synergistic one-dimensional nanofibers as heterostructure absorbers for tunable and efficient microwave absorption. Nano-Micro Lett. **15**, 13 (2023). 10.1007/s40820-022-00986-3 10.1007/s40820-022-00986-3 PMC9755410 36520259

[13] Y.L. Zhang, K.P. Ruan, K. Zhou, J.W. Gu, Controlled distributed Ti_3_C_2_T_x_ hollow microspheres on thermally conductive polyimide composite films for excellent electromagnetic interference shielding. Adv. Mater. **35**(16), 2211642 (2023). 10.1002/adma.202211642 10.1002/adma.202211642 36703618

[14] F. Wu, M.Y. Ling, L.Y. Wan, P. Liu, Y.B. Wang et al., Three-dimensional FeMZn (M = Co or Ni) MOFs: ions coordinated self-assembling processes and boosting microwave absorption. Chem. Eng. J. **435**, 134905 (2022). 10.1016/j.cej.2022.134905

[15] Y.F. He, Q. Su, D.D. Liu, L. Xia, X.X. Huang et al., Surface engineering strategy for MXene to tailor electromagnetic wave absorption performance. Chem. Eng. J. **491**, 152041 (2024). 10.1016/j.cej.2024.152041

[16] H.L. Lv, Z.H. Yang, H.G. Pan, R.B. Wu, Electromagnetic absorption materials: current progress and new frontiers. Prog. Mater. Sci. **127**, 100946 (2022). 10.1016/j.pmatsci.2022.100946

[17] H. Zhao, T. Gao, J. Yun, L.X. Chen, Robust liquid metal reinforced cellulose nanofiber/MXene composite film with Janus structure for electromagnetic interference shielding and electro-/photothermal conversion applications. J. Mater. Sci. Technol. **191**, 23–32 (2024). 10.1016/j.jmst.2023.12.035

[18] J.M. Yang, H. Wang, Y.L. Zhang, H.X. Zhang, J.W. Gu, Layered structural PBAT composite foams for efficient electromagnetic interference shielding. Nano-Micro Lett. **16**, 31 (2024). 10.1007/s40820-023-01246-8 10.1007/s40820-023-01246-8 PMC10667195 37994969

[19] L.Y. Liang, Q.M. Li, X. Yan, Y.Z. Feng, Y.M. Wang et al., Multifunctional magnetic Ti_3_C_2_T_x_ MXene/graphene aerogel with superior electromagnetic wave absorption performance. ACS Nano **15**(4), 6622–6632 (2021). 10.1021/acsnano.0c09982 33780231 10.1021/acsnano.0c09982

[20] J. Wang, Z. Jia, X. Liu, J. Dou, B. Xu, B. Wang, G. Wu, Construction of 1D heterostructure NiCo@C/ZnO nanorod with enhanced microwave absorption. Nano-Micro Lett. **13**, 175 (2021). 10.1007/s40820-021-00704-5 10.1007/s40820-021-00704-5 PMC8368508 34398334

[21] C.H. Wei, L.Z. Shi, M.Q. Li, M.K. He, M.J. Li et al., Hollow engineering of sandwich NC@Co/NC@MnO_2_ composites toward strong wideband electromagnetic wave attenuation. J. Mater. Sci. Technol. **175**, 194–203 (2024). 10.1016/j.jmst.2023.08.020

[22] J.C. Shu, M.S. Cao, Y.L. Zhang, Y.Z. Wang, Q.L. Zhao et al., Atomic-molecular engineering tailoring graphene microlaminates to tune multifunctional antennas. Adv. Funct. Mater. **33**(15), 2212379 (2023). 10.1002/adfm.202212379

[23] L.H. Yao, Y.C. Wang, J.G. Zhao, Y.Q. Zhu, M.S. Cao, Multifunctional nanocrystalline-assembled porous hierarchical material and device for integrating microwave absorption, electromagnetic interference shielding, and energy storage. Small **19**(25), 2208101 (2023). 10.1002/smll.202208101 10.1002/smll.202208101 36932880

[24] D.L. Tan, Q. Wang, M.R. Li, L.M. Song, F. Zhang et al., Magnetic media synergistic carbon fiber@Ni/NiO composites for high-efficiency electromagnetic wave absorption. Chem. Eng. J. **492**, 152245 (2024). 10.1016/j.cej.2024.152245

[25] A.L. Feng, L. Liu, P.T. Liu, Y.Q. Zu, F.B. Han et al., Interfacial nanoparticles of Co_2_P/Co_3_Fe_7_ encapsulated in N-doped carbon nanotubes as bifunctional oxygen electrocatalysts for rechargeable zinc-air batteries. Mater. Today Energy **44**, 101626 (2024). 10.1016/j.mtener.2024.101626

[26] F.H. Yu, P.F. Jia, L. Song, Y. Hu, B.B. Wang et al., Multifunctional fabrics based on copper sulfide with excellent electromagnetic interference shielding performance for medical electronics and physical therapy. Chem. Eng. J. **472**, 145091 (2023). 10.1016/j.cej.2023.145091

[27] Y.Q. Zhou, L.F. Zhang, H.L. Suo, W.B. Hua, S. Indris et al., Atomic cobalt vacancy-cluster enabling optimized electronic structure for efficient water splitting. Adv. Funct. Mater. **31**(26), 2101797 (2021). 10.1002/adfm.202101797

[28] Y. Han, M.J. Han, T.B. Zhao, Z.H. Xia, J.X. Zou et al., Design of morphology-controlled cobalt-based spinel oxides for efficient X-band microwave absorption. Mater. Res. Bull. **172**, 112670 (2024). 10.1016/j.materresbull.2023.112670

[29] J.Q. Zeng, P.F. Qi, Y. Wang, Y.H. Liu, K.Y. Sui, Electrostatic assembly construction of polysaccharide functionalized hybrid membrane for enhanced antimony removal. J. Hazard. Mater. **410**, 124633 (2021). 10.1016/j.jhazmat.2020.124633 33243653 10.1016/j.jhazmat.2020.124633

[30] M.Y. Yuan, B. Zhao, C.D. Yang, K. Pei, L.Y. Wang et al., Remarkable magnetic exchange coupling via constructing bi-magnetic interface for broadband lower-frequency microwave absorption. Adv. Funct. Mater. **32**(33), 1103161 (2022). 10.1002/adfm.202203161

[31] X.M. Huang, X.H. Liu, Y. Zhang, J.X. Zhou, G.L. Wu et al., Construction of NiCeO_x_ nanosheets-skeleton cross-linked by carbon nanotubes networks for efficient electromagnetic wave absorption. J. Mater. Sci. Technol. **147**, 16–25 (2023). 10.1016/j.jmst.2022.12.001

[32] H.P. Lv, C. Wu, J. Tang, H.F. Du, F.X. Qin et al., Two-dimensional SnO/SnO_2_ heterojunctions for electromagnetic wave absorption. Chem. Eng. J. **411**, 128445 (2021). 10.1016/j.cej.2021.128445

[33] T.T. Zheng, Y. Zhang, Z.R. Jia, J.H. Zhu, G.L. Wu et al., Customized dielectric-magnetic balance enhanced electromagnetic wave absorption performance in Cu_x_S/CoFe_2_O_4_ composites. Chem. Eng. J. **457**, 140876 (2023). 10.1016/j.cej.2022.140876

[34] D. Lan, Y. Hu, M. Wang, Y. Wang, Z.G. Gao et al., Perspective of electromagnetic wave absorbing materials with continuously tunable effective absorption frequency bands. Compos. Commun. **50**, 101993 (2024). 10.1016/j.coco.2024.101993

[35] X.L. Cao, X.H. Liu, J.H. Zhu, Z.R. Jia, J.K. Liu et al., Optimal particle distribution induced interfacial polarization in hollow double-shell composites for electromagnetic waves absorption performance. J Colloid Interf. Sci. **634**, 268–278 (2023). 10.1016/j.jcis.2022.12.048 10.1016/j.jcis.2022.12.048 36535164

[36] T. Zhao, Z. Jia, J. Liu, Y. Zhang, G. Wu, P. Yin, Multiphase interfacial regulation based on hierarchical porous molybdenum selenide to build anticorrosive and multiband tailorable absorbers. Nano-Micro Lett. **16**, 6 (2024). 10.1007/s40820-023-01212-4 10.1007/s40820-023-01212-4 PMC10627983 37930594

[37] Z.C. Wu, H.W. Cheng, C. Jin, B.T. Yang, C.Y. Xu et al., Dimensional design and core-shell engineering of nanomaterials for electromagnetic wave absorption. Adv. Mater. **34**(11), 2107538 (2022). 10.1002/adma.202107538 10.1002/adma.202107538 34755916

[38] Z.H. Zhou, Q.Q. Zhu, Y. Liu, Y. Zhang, Z.R. Jia et al., Construction of self-assembly based tunable absorber: lightweight, hydrophobic and self-cleaning properties. Nano-Micro Lett. **15**, 137 (2023). 10.1007/s40820-023-01108-3 10.1007/s40820-023-01108-3 PMC10225461 37245198

[39] J.L. Liu, H.S. Liang, B. Wei, J.J. Yun, L.M. Zhang et al., “Matryoshka Doll” heterostructures induce electromagnetic parameters fluctuation to tailor electromagnetic wave absorption. Small Struct. **4**(7), 2200379 (2023). 10.1002/sstr.202200379

[40] X.L. Chen, F. Zhang, D. Lan, S.J. Zhang, S.X. Du et al., State-of-the-art synthesis strategy for nitrogen-doped carbon-based electromagnetic wave absorbers: from the perspective of nitrogen source. Adv. Compos. Hybrid Mater. **6**(6), 220 (2023). 10.1007/s42114-023-00792-4

[41] S. Zhang, X.H. Liu, C.Y. Jia, Z.S. Sun, H.W. Jiang et al., Integration of multiple heterointerfaces in a hierarchical 0D@2D@1D structure for lightweight, flexible, and hydrophobic multifunctional electromagnetic protective fabrics. Nano-Micro Lett. **15**, 204 (2023). 10.1007/s40820-023-01179-2 10.1007/s40820-023-01179-2 PMC10457279 37624447

[42] J.J. Li, D. Lan, Y.H. Cheng, Z.R. Jia, P.B. Liu et al., Constructing mixed-dimensional lightweight magnetic cobalt-based composites heterostructures: an effective strategy to achieve boosted microwave absorption and self-anticorrosion. J. Mater. Sci. Technol. **196**, 60–70 (2024). 10.1016/j.jmst.2024.02.016

[43] M.J. Han, D. Lan, Z.M. Zhang, Y.Z. Zhao, J.X. Zou et al., Micro-sized hexapod-like CuS/Cu_9_S_5_ hybrid with broadband electromagnetic wave absorption. J. Mater. Sci. Technol. **214**, 302–312 (2025). 10.1016/j.jmst.2024.07.014

[44] G.J. Ma, D. Lan, Y. Zhang, X.Y. Sun, Z.R. Jia et al., Microporous cobalt ferrite with bio-carbon loosely decorated to construct multi-functional composite for dye adsorption, anti-bacteria and electromagnetic protection. Small **20**, 2404449 (2024). 10.1002/smll.202404449 10.1002/smll.202404449 39011980

[45] Z. Li, J. Ding, H.L. Wang, K. Cui, T. Stephenson et al., High rate SnO_2_-graphene dual aerogel anodes and their kinetics of lithiation and sodiation. Nano Energy **15**, 369–378 (2015). 10.1016/j.nanoen.2015.04.018

[46] L. Wu, H.Y. Lu, L.F. Xiao, J.F. Qian, X.P. Ai et al., A tin(II) sulfide-carbon anode material based on combined conversion and alloying reactions for sodium-ion batteries. J. Mater. Chem. A **2**(39), 16424–16428 (2014). 10.1039/c4ta03365e

[47] Y. Zheng, T.F. Zhou, C.F. Zhang, J.F. Mao, H.K. Liu et al., Boosted charge transfer in sns/sno_2_ heterostructures: toward high rate capability for sodium-ion batteries. Angew. Chem. Int. Ed. **55**(10), 3408–3413 (2016). 10.1002/anie.201510978 10.1002/anie.201510978 26844806

[48] Y. Han, D. Lan, M.J. Han, Z.H. Xia, J.X. Zou et al., Construction of flower-like MoS_2_ decorated on Cu doped CoZn–ZIF derived N-doped carbon as superior microwave absorber. Nano Res. **17**(9), 8250–8260 (2024). 10.1007/s12274-024-6859-z

[49] Z.H. Ma, K. Yang, D. Li, H. Liu, S.C. Hui et al., The electron migration polarization boosting electromagnetic wave absorption based on Ce atoms modulated yolk@shell Fe_x_N@NGC. Adv. Mater. **36**(23), 2314233 (2024). 10.1002/adma.202314233 10.1002/adma.202314233 38380795

[50] Z.H. Zhou, X.F. Zhou, D. Lan, Y. Zhang, Z.R. Jia et al., Modulation engineering of electromagnetic wave absorption performance of layered double hydroxides derived hollow metal carbides integrating corrosion protection. Small **20**, 2305849 (2024). 10.1002/smll.202305849 10.1002/smll.202305849 37817350

[51] Y. Gao, J.J. Lin, X. Chen, Z.D. Tang, G. Qin et al., Engineering 2D MXene and LDH into 3D hollow framework for boosting photothermal energy storage and microwave absorption. Small **19**(49), 2303113 (2023). 10.1002/smll.202303113 10.1002/smll.202303113 37605334

[52] P.K. Wu, X.K. Kong, Y.R. Feng, W. Ding, Z.G. Sheng et al., Phase engineering on amorphous/crystalline γ-Fe_2_O_3_ nanosheets for boosting dielectric loss and high-performance microwave absorption. Adv. Funct. Mater. **34**(10), 2311983 (2024). 10.1002/adfm.202311983

[53] R. Barik, V. Tanwar, R. Kumar, P.P. Ingole, A high energy density and high rate capability flexible supercapacitor based on electro-spun highly porous SnO_2_@carbon nanofibers. J. Mater. Chem. A **8**(30), 15110–15121 (2020). 10.1039/d0ta04355a

[54] F.S. Yuan, Y. Huang, J.S. Qian, M.M. Rahman, P.M. Ajayan et al., Free-standing SnS/carbonized cellulose film as durable anode for lithium-ion batteries. Carbohyd. Polym. **255**, 117400 (2021). 10.1016/j.carbpol.2020.117400 10.1016/j.carbpol.2020.117400 33436227

[55] J.K. Liu, Z.R. Jia, Y.H. Dong, J.J. Li, X.L. Cao et al., Structural engineering and compositional manipulation for high-efficiency electromagnetic microwave absorption. Mater. Today Phys. **27**, 100801 (2022). 10.1016/j.mtphys.2022.100801

[56] K.Y. Hu, C. Ming, Y.T. Liu, C. Zheng, S.N. Zhang et al., Introducing sulfur vacancies and in-plane SnS_2_/SnO_2_ heterojunction in SnS_2_ nanosheets to promote photocatalytic activity. Chin. Chem. Lett. **31**(10), 2809–2813 (2020). 10.1016/j.cclet.2020.07.052

[57] K.Y. Hu, C. Ming, Y.T. Liu, C. Zheng, S.N. Zhang et al., Introducing sulfur vacancies and in-plane SnS_2_/SnO_2_ heterojunction in SnS_2_ nanosheets to promote photocatalytic activity. Chin. Chem. Lett. **31**, 2809–2813 (2020). 10.1016/j.cclet.2020.07.052

[58] L.G. Li, Z.C. Chai, W. Jin, H. Sun, J.H. He et al., Sulfur vacancy in SnS_2_ nanoflake adjusted by precursor and improved photocatalytic performance. J. Alloy. Compd. **932**, 167658 (2023). 10.1016/j.jallcom.2022.167658

[59] Y.X. Qin, Z.J. Wei, Y.N. Bai, Effect of vacancy defects of SnS on gas adsorption and its potential for selective gas detection. Vacuum **183**, 109792 (2021). 10.1016/j.vacuum.2020.109792

[60] J.B. Wang, J.J. Huang, S.P. Huang, H. Notohara, K. Urita et al., Rational design of hierarchical SnS_2_ microspheres with S vacancy for enhanced sodium storage performance. ACS Sustain. Chem. Eng. **8**(25), 9519–9525 (2020). 10.1021/acssuschemeng.0c02535

[61] Y.X. Qin, S.H. Chen, Y.N. Bai, Adsorption and sensing performance toward methanol vapor on SnS/SnS_2_ in-plane heterostructures. ACS Appl. Electron. Mater. **4**(1), 158–167 (2022). 10.1021/acsaelm.1c00911

[62] J.L. Liu, L.M. Zhang, H.J. Wu, Enhancing the low/middle-frequency electromagnetic wave absorption of metal sulfides through F-regulation engineering. Adv. Funct. Mater. **32**(13), 2110469 (2021). 10.1002/adfm.202110496

[63] X.X. Chu, Y.Y. Liao, L. Wang, J.R. Li, H. Xu, Engineering sulfur vacancies for boosting electrocatalytic reactions. Chin. Chem. Lett. **34**, 108285 (2023). 10.1016/j.cclet.2023.108285

[64] X.L. Cao, D. Lan, Y. Zhang, Z.R. Jia, G.L. Wu et al., Construction of three-dimensional conductive network and heterogeneous interfaces via different ratio for tunable microwave absorption. Adv. Compos. Hybrid Mater. **6**, 187 (2023). 10.1007/s42114-023-00763-9

[65] Y. Liu, D.L. Pan, M.W. Xiong, Y. Tao, X.F. Chen et al., In-situ fabrication SnO_2_/SnS_2_ heterostructure for boosting the photocatalytic degradation of pollutants. Chin. J. Catal. **41**(10), 1554–1563 (2020). 10.1016/S1872-2067(19)63498-4

[66] Z.M. Tang, L. Xu, C. Xie, L.R. Guo, L.B. Zhang et al., Synthesis of CuCo_2_S_4_@expanded graphite with crystal/amorphous heterointerface and defects for electromagnetic wave absorption. Nat. Commun. **14**(1), 5951 (2023). 10.1038/s41467-023-41697-6 37741860 10.1038/s41467-023-41697-6 PMC10517935

[67] S.J. Zhang, D. Lan, J.J. Zheng, X.L. Chen, A.L. Feng et al., Rational construction of heterointerfaces in biomass sugarcane-derived carbon for superior electromagnetic wave absorption. Int. J. Miner. Metall. Mater. (2024). 10.1007/s12613-024-2875-y

[68] Y.Y. Lian, D. Lan, X.D. Jiang, L. Wang, S. Yan et al., Multifunctional electromagnetic wave absorbing carbon fiber/Ti_3_C_2_T_X_ MXene fabric with superior near-infrared laser dependent photothermal antibacterial behaviors. J. Colloid Interf. Sci. **676**, 217–226 (2024). 10.1016/j.jcis.2024.07.102 10.1016/j.jcis.2024.07.102 39024822

[69] J.X. Xiao, B.B. Zhan, M.K. He, X.S. Qi, X. Gong et al., Interfacial polarization loss improvement induced by the hollow engineering of necklace-like PAN/carbon nanofibers for boosted microwave absorption. Adv. Funct. Mater. **34**, 2316722 (2024). 10.1002/adfm.202316722

[70] J.H. Wen, D. Lan, Y.Q. Wang, L.G. Ren, A.L. Feng et al., Absorption properties and mechanism of lightweight and broadband electromagnetic wave absorbing porous carbon by swelling treatment. Int. J. Miner. Metall. Mater. **31**, 1701–1712 (2024). 10.1007/s12613-024-2881-0

[71] M.Y. Yuan, H.L. Lv, H.W. Cheng, B. Zhao, G.Y. Chen et al., Atomic and electronic reconstruction in defective 0D molybdenum carbide heterostructure for regulating lower-frequency microwaves. Adv. Funct. Mater. **33**(33), 2302003 (2023). 10.1002/adfm.202302003

[72] P.F. Yin, D. Lan, C.F. Lu, Z.R. Jia, A.L. Feng et al., Research progress of structural regulation and composition optimization to strengthen absorbing mechanism in emerging composites for efficient electromagnetic protection. J. Mater. Sci. Technol. **204**, 204–223 (2025). 10.1016/j.jmst.2024.04.007

[73] Z.W. Hao, J. Zhou, S.N. Lin, D. Lan, H.Y. Li et al., Customized heterostructure of transition metal carbides as high-efficiency and anti-corrosion electromagnetic absorbers. Carbon **228**, 119323 (2024). 10.1016/j.carbon.2024.119323

[74] R.Y. Tan, Y.J. Liu, W.J. Li, J.T. Zhou, P. Chen et al., Multi-scale dispersion engineering on biomass-derived materials for ultra-wideband and wide-angle microwave absorption. Adv. Funct. Mater. **34**, 2301772 (2024). 10.1002/smtd.202301772 10.1002/smtd.202301772 38513234

[75] J.X. Zhou, D. Lan, F. Zhang, Y.H. Cheng, Z.R. Jia et al., Self-assembled MoS_2_ cladding for corrosion resistant and frequency-modulated electromagnetic wave absorption materials from X-band to Ku-band. Small **19**(52), 2304932 (2023). 10.1002/smll.202304932 10.1002/smll.202304932 37635102

[76] N.N. Wu, B.B. Zhao, Y.Y. Lian, S.S. Liu, Y. Xian et al., Metal organic frameworks derived Ni_x_Se_y_@NC hollow microspheres with modifiable composition and broadband microwave attenuation. Carbon **226**, 119215 (2024). 10.1016/j.carbon.2024.119215

[77] J.H. Wang, L. Zhang, J.F. Yan, J.N. Yun, W. Zhao et al., MXene-based ultrathin electromagnetic wave absorber with hydrophobicity, anticorrosion, and quantitively classified electrical losses by intercalation growth nucleation engineering. Adv. Funct. Mater. **34**, 2402419 (2024). 10.1002/adfm.202402419

[78] X.L. Chen, D. Lan, L.T. Zhou, Z. Zeng, Y.K. Liu et al., Rational construction of ZnFe_2_O_4_ decorated hollow carbon cloth towards effective electromagnetic wave absorption. Ceram. Int. **50**, 24549–24557 (2024). 10.1016/j.ceramint.2024.04.190

[79] Y. Liu, X.F. Zhou, Z.R. Jia, H.J. Wu, G.L. Wu, Oxygen vacancy induced dielectric polarization prevails in electromagnetic wave absorbing mechanism for Mn-based MOFs-derived composites. Adv. Funct. Mater. **32**(34), 2204499 (2022). 10.1002/adfm.202204499

[80] Z.R. Jia, J.K. Liu, Z.G. Gao, C.H. Zhang, G.L. Wu, Molecular intercalation-induced two-phase evolution engineering of 1T and 2H–MS_2_ (M=Mo, V, W) for interface-polarization-enhanced electromagnetic absorbers. Adv. Funct. Mater. **34**, 2405523 (2024). 10.1002/adfm.202405523

[81] T. Liu, Y.N. Zhang, C. Wang, Y.F. Kang, M. Wang et al., Multifunctional MoC_x_ hybrid polyimide aerogel with modified porous defect engineering for highly efficient electromagnetic wave absorption. Small **20**, 2308378 (2024). 10.1002/smll.202308378 10.1002/smll.202308378 38453681

[82] H.B. Zhang, J.Y. Cheng, H.H. Wang, Z.H. Huang, Q.B. Zheng et al., Initiating VB-group laminated NbS_2_ electromagnetic wave absorber toward superior absorption bandwidth as large as 6.48 GHz through phase engineering modulation. Adv. Funct. Mater. **32**(6), 2108194 (2022). 10.1002/adfm.202108194

[83] Z.G. Gao, D. Lan, X.Y. Ren, Z.R. Jia, G.L. Wu, Manipulating cellulose-based dual-network coordination for enhanced electromagnetic wave absorption in magnetic porous carbon nanocomposites. Compos. Commun. **48**, 101922 (2024). 10.1016/j.coco.2024.101922

[84] Z.Y. Shen, D. Lan, Y. Cong, Y.Y. Lian, N.N. Wu et al., Tailored heterogeneous interface based on porous hollow In–Co–C nanorods to construct adjustable multi-band microwave absorber. J. Mater. Sci. Technol. **181**, 128–137 (2024). 10.1016/j.jmst.2023.10.007

[85] H.S. Liang, L.M. Zhang, H.J. Wu, Exploration of twin-modified grain boundary engineering in metallic copper predominated electromagnetic wave absorber. Small **18**(38), 2203620 (2022). 10.1002/smll.202203620 10.1002/smll.202203620 35989098

[86] Q.L. Zhang, D. Lan, S.L. Deng, J.W. Gu, Y.Q. Wang et al., Constructing multiple heterogeneous interfaces in one-dimensional carbon fiber materials for superior electromagnetic wave absorption. Carbon **226**, 119233 (2024). 10.1016/j.carbon.2024.119233

[87] Z.G. Gao, A. Iqbal, T. Hassan, S.C. Hui, H.J. Wu et al., Tailoring built-in electric field in a self-assembled zeolitic imidazolate framework/MXene nanocomposites for microwave absorption. Adv. Mater. **36**(19), 2311411 (2024). 10.1002/adma.202311411 10.1002/adma.202311411 38288859

[88] L.L. Liang, W.H. Gu, Y. Wu, B.S. Zhang, G.H. Wang et al., Heterointerface engineering in electromagnetic absorbers: new insights and opportunities. Adv. Mater. **34**(4), 2106195 (2022). 10.1002/adma.202106195 10.1002/adma.202106195 34599773

[89] F. Pan, K. Pei, G. Chen, H.T. Guo, H.J. Jiang et al., Integrated electromagnetic device with on-off heterointerface for intelligent switching between wave-absorption and wave-transmission. Adv. Funct. Mater. **33**(49), 2306599 (2023). 10.1002/adfm.202306599

[90] J. Yan, Z.D. Ye, D. Lan, W.X. Chen, Z.R. Jia et al., Transition metal carbides towards electromagnetic wave absorption application: state of the art and perspectives. Compos. Commun. **48**, 101954 (2024). 10.1016/j.coco.2024.101954

[91] L.Y. Yuan, W.X. Zhao, Y.K. Miao, C. Wang, A.G. Cui et al., Constructing core-shell carbon fiber/polypyrrole/CoFe_2_O_4_ nanocomposite with optimized conductive loss and polarization loss toward efficient electromagnetic absorption. Adv. Compos. Hybrid Mater. **7**(2), 70 (2024). 10.1007/s42114-024-00864-z

[92] S. Zhang, Z.R. Jia, Y. Zhang, G.L. Wu, Electrospun Fe_0.64_Ni_0.36_/MXene/CNFs nanofibrous membranes with multicomponent heterostructures as flexible electromagnetic wave absorbers. Nano Res. **16**, 3395–3407 (2023). 10.1007/s12274-022-5368-1

[93] W.J. Li, W.C. Li, Z.B. Ma, Y. Kang, T. Zou et al., N atoms regulate heterogeneous crystal phase engineering of mesoporous magnetic Fe_x_N nanofibers to promote electromagnetic wave dissipation of magnetic composite aerogel absorbers. Chem. Eng. J. **481**, 148584 (2024). 10.1016/j.cej.2024.148584

[94] W.B. Deng, T.H. Li, H. Li, J. Abdul, L.T. Liu et al., MOF derivatives with gradient structure anchored on carbon foam for high-performance electromagnetic wave absorption. Small **20**, 2329806 (2024). 10.1002/smll.202309806 10.1002/smll.202309806 38243852

[95] C.J. Wang, H.T. Jiang, X.Z. Cao, X. He, X.B. Chen et al., Graphite wrapped FeNi_3_/Co with carbon nanotubes anchored on MgO@carbon fiber reinforcements via continuous fabrication for high-efficiency microwave attenuation. Adv. Fiber Mater. (2024). 10.1007/s42765-024-00446-0

[96] J.L. Gao, L. Chang, B. Ni, X.C. Zhang, L. Li et al., Dielectric modulation engineering in hierarchically ordered porous Ti_3_C_2_T_x_ MXene/rhenium disulfide aerogel toward potential electromagnetic wave absorption and infrared stealth. Adv. Compos. Hybrid Mater. **7**(3), 103 (2024). 10.1007/s42114-024-00917-3

[97] B.J. Wang, W. Wei, F.Z. Huang, F.H. Liu, S.K. Li et al., Orbital hybridization induced dipole polarization and room temperature magnetism of atomic Co–N_4_–C toward electromagnetic energy attenuation. Adv. Funct. Mater. **34**, 2404484 (2024). 10.1002/adfm.202404484

[98] X.J. Zeng, X. Jiang, Y. Ning, Y.F. Gao, R.C. Che, Constructing built-in electric fields with semiconductor junctions and Schottky junctions based on mo–mxene/mo-metal sulfides for electromagnetic response. Nano-Micro Lett. **16**, 213 (2024). 10.1007/s40820-024-01449-7 10.1007/s40820-024-01449-7 PMC11166625 38861114

[99] B. Shan, Y. Wang, X.Y. Ji, Y. Huang, Enhancing low-frequency microwave absorption through structural polarization modulation of MXenes. Nano-Micro Lett. **16**, 212 (2024). 10.1007/s40820-024-01437-x 10.1007/s40820-024-01437-x PMC11166627 38861180

[100] Y.T. Qian, Z.C. Wu, X.W. Lv, M.Q. Huang, L.J. Rao, Fixed-point atomic regulation engineered low-thickness wideband microwave absorption. Small **20**, 2401878 (2024). 10.1002/smll.202401878 10.1002/smll.202401878 38742982

[101] C.Y. Xu, K.C. Luo, Y.Q. Du, H.B. Zhang, X.W. Lv et al., Anisotropic interfaces support the confined growth of magnetic nanometer-sized heterostructures for electromagnetic wave absorption. Adv. Funct. Mater. **33**(47), 2307529 (2023). 10.1002/adfm.202307529

[102] M.Q. Huang, L. Wang, K. Pei, B.X. Li, W.B. You et al., Heterogeneous interface engineering of Bi-Metal MOFs-derived ZnFe_2_O_4_–ZnO–Fe@C microspheres via confined growth strategy toward superior electromagnetic wave absorption. Adv. Funct. Mater. **33**(3), 2308898 (2023). 10.1002/adfm.202308898

[103] Y.H. Ge, H.G. Wang, T.Q. Wu, B. Hu, Y.Z. Shao et al., Accordion-like reduced graphene oxide embedded with Fe nanoparticles between layers for tunable and broadband electromagnetic wave absorption. J. Colloid Interf. Sci. **628**, 1019–1030 (2022). 10.1016/j.jcis.2022.08.020 10.1016/j.jcis.2022.08.020 36049278

[104] Y.J. Liu, X.F. Wei, X.X. He, J.R. Yao, R.Y. Tan et al., Multifunctional shape memory composites for Joule heating, self-healing, and highly efficient microwave absorption. Adv. Funct. Mater. **33**(5), 2211352 (2023). 10.1002/adfm.202211352

[105] Y. Zhang, D. Lan, T. Hou, M. Jia, Z. Jia, J. Gu, G. Wu, Multifunctional electromagnetic wave absorbing carbon fiber/Ti_3_C_2_T_X_ MXene fabric with ultra-wide absorption band. Carbon **230**, 119594 (2024). 10.1016/j.carbon.2024.119594

[106] X.F. Xu, S.H. Shi, Y.L. Tang, G.Z. Wang, M.F. Zhou et al., Growth of NiAl-layered double hydroxide on graphene toward excellent anticorrosive microwave absorption application. Adv. Sci. **8**, 2002658 (2021). 10.1002/advs.202002658 10.1002/advs.202002658 PMC7927622 33717840

[107] J.H. Zhu, D. Lan, X.H. Liu, S.H. Zhang, Z.R. Jia et al., Porous structure fibers based on multi-element heterogeneous components for optimized electromagnetic wave absorption and self-anticorrosion performance. Small (2024). 10.1002/smll.202403689 10.1002/smll.202403689 39128133

[108] D. Lan, H.F. Li, M. Wang, Y.J. Ren, J. Zhang et al., Recent advances in construction strategies and multifunctional properties of flexible electromagnetic wave absorbing materials. Mater. Res. Bull. **171**, 112630 (2024). 10.1016/j.materresbull.2023.112630

[109] Y. Cheng, D. Lan, Z. Jia, Z. Gao, X. Liu et al., MOF derivatives anchored to multichannel hollow carbon fibers with gradient structures for corrosion resistance and efficient electromagnetic wave absorption. J. Mater. Sci. Technol. (2024). 10.1016/j.jmst.2024.08.004

[110] H.L. Lv, Y.X. Yao, S.C. Li, G.L. Wu, B. Zhao et al., Staggered circular nanoporous graphene converts electromagnetic waves into electricity. Nat. Commun. **14**, 1982 (2023). 10.1016/10.1038/s41467-023-37436-6 37031210 10.1038/s41467-023-37436-6 PMC10082851

[111] M.J. Cui, X.Y. Chen, S.X. Mei, S.M. Ren, Bioinspired polydopamine nanosheets for the enhancement in anti-corrosion performance of water-borne epoxy coatings. Chem. Eng. J. **471**, 144760 (2023). 10.1016/j.cej.2023.144760

[112] X.B. Xie, H.H. Wang, H. Kimura, C. Ni, W. Du et al., NiCoZn/C@melamine sponge-derived carbon composites with high-performance electromagnetic wave absorption. Int. J. Miner. Metall. Mater. **31**(10), 2274–2286 (2024). 10.1007/s12613-024-2880-1

[113] J. Zhou, X. Huang, D. Lan, Y. Cheng, F. Xue, C. Jia et al., Polymorphic cerium-based prussian blue derivatives with in situ growing CNT/Co heterojunctions for enhanced microwave absorption via polarization and magnetization. Nano Res. **17**(3), 2050–2060 (2024). 10.1002/smll.202304932

[114] X.B. Zhu, Q.Q. Yan, L. Cheng, H. Wu, H.C. Zha et al., Self-alignment of cationic graphene oxide nanosheets for anticorrosive reinforcement of epoxy coatings. Chem. Eng. J. **389**, 124435 (2020). 10.1016/j.cej.2021140.124435

[115] J. Jiang, D. Lan, Y. Li, J. Yang, S. Deng, Q. He, Y. Wang, Construction of spherical heterogeneous interface on ZnFe_2_O_4_@C composite nanofibers for highly efficient microwave absorption. Ceram. Int. (2024). 10.1016/j.ceramint.2024.07.197

[116] H.L. Lv, Z.H. Yang, B. Liu, G.L. Wu, Z.C. Lou et al., A flexible electromagnetic wave-electricitiy harvester. Nat. Commun. **12**(1), 834 (2021). 10.1038/s41467-021-21103-9 33547310 10.1038/s41467-021-21103-9 PMC7864982

[117] Y. Dong, D. Lan, S. Xu, J. Gu, Z. Jia, G. Wu, Controllable fiberization engineering of cobalt anchored mesoporous hollow carbon spheres for positive feedback to electromagnetic wave absorption. Carbon **228**, 119339 (2024). 10.1016/j.carbon.2024.119339

[118] Z. Jia, L. Sun, Z. Gao, D. Lan, Modulating magnetic interface layer on porous carbon heterostructures for efficient microwave absorption. Nano Res. (2024). 10.1007/s12274-024-6939-0

[119] J.Q. Tao, L.L. Xu, C.B. Pei, Y.S. Gu, Y.R. He et al., Catfish effect induced by anion sequential doping for microwave absorption. Adv. Funct. Mater. **33**(8), 2211996 (2023). 10.1002/adfm.202211996

[120] T. Zhao, D. Lan, Z. Jia, Z. Gao, G. Wu, Hierarchical porous molybdenum carbide synergic morphological engineering towards broad multi-band tunable microwave absorption. Nano Res. (2024). 10.1007/s12274-024-6938-1

[121] C.L. Zhou, Z. Li, J. Li, T.C. Yuan, B. Chen et al., Epoxy composite coating with excellent anticorrosion and self-healing performances based on multifunctional zeolitic imidazolate framework derived nanocontainers. Chem. Eng. J. **385**, 123835 (2020). 10.1016/j.cej.2019.123835

