# Supplementary material for: Multiple Tin Compounds Modified Carbon Fibers to Construct Heterogeneous Interfaces for Corrosion Prevention and Electromagnetic Wave Absorption
Source: Nanomicro Lett. 2024 Sep 27;17:23. doi: 10.1007/s40820-024-01527-w (PMC11436513; doi:10.1007/s40820-024-01527-w)
Supplement: Supplementary file 1 — Supplementary file1 (DOCX 7199 kb) [file 40820_2024_1527_MOESM1_ESM.docx]

Supporting Information for

**Multiple Tin Compounds Modified Carbon Fibers to Construct Heterogeneous Interfaces for Corrosion Prevention and Electromagnetic Wave Absorption**

Zhiqiang Guo^1,3^, Di Lan^2^, Zirui Jia^3,*^, Zhenguo Gao^1^, Xuetao Shi^4^, Mukun He^4^, Hua Guo^4^, Guanglei Wu^3,*^, Pengfei Yin^1,*^

^1^College of Science, Sichuan Agricultural University, Ya’an 625014, P. R. China

^2^School of Materials Science and Engineering, Hubei University of Automotive Technology, Shiyan 442002, P. R. China

^3^Institute of Materials for Energy and Environment, State Key Laboratory of Bio-fibers and Eco-textiles, College of Materials Science and Engineering, Qingdao University, Qingdao 266071, P. R. China

^4^Shaanxi Key Laboratory of Macromolecular Science and Technology, School of Chemistry and Chemical Engineering, Northwestern Polytechnical University, Xi’an, Shaanxi 710072, P. R. China

*Corresponding authors. E-mail: [jiazirui@qdu.edu.cn](mailto:jiazirui@qdu.edu.cn) (Zirui Jia); [wuguanglei@qdu.edu.cn](mailto:wuguanglei@qdu.edu.cn) (Guanglei Wu); [yinpengfei@sicau.edu.cn](mailto:yinpengfei@sicau.edu.cn) (Pengfei Yin)

**Supplementary Figures and Tables**


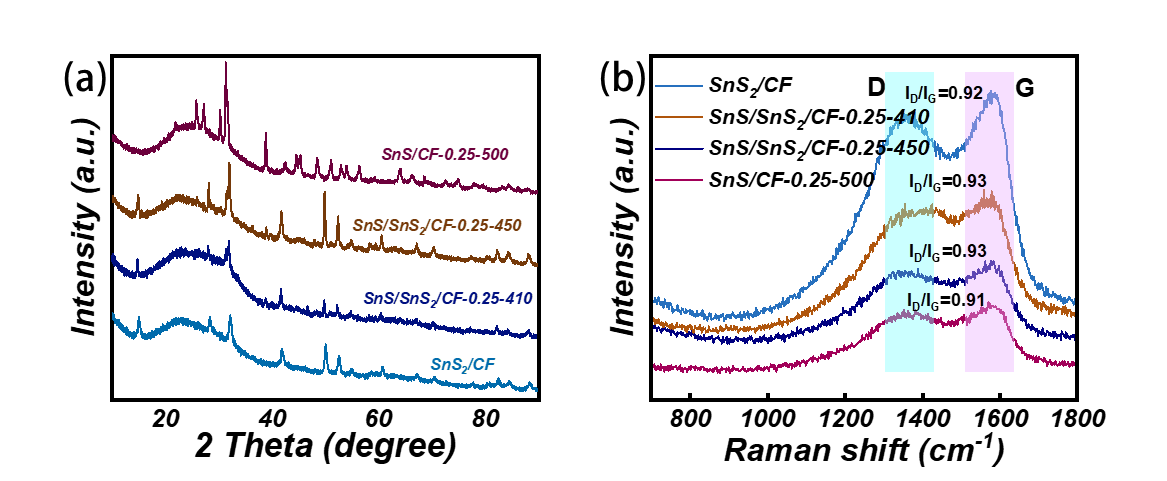


**Fig. S1** **a** XRD image and **b** Raman image of SnS_2_/CF, SnS/SnS_2_/CF-0.25-410, SnS/SnS_2_/CF-0.25-450, and SnS/CF-0.25-500


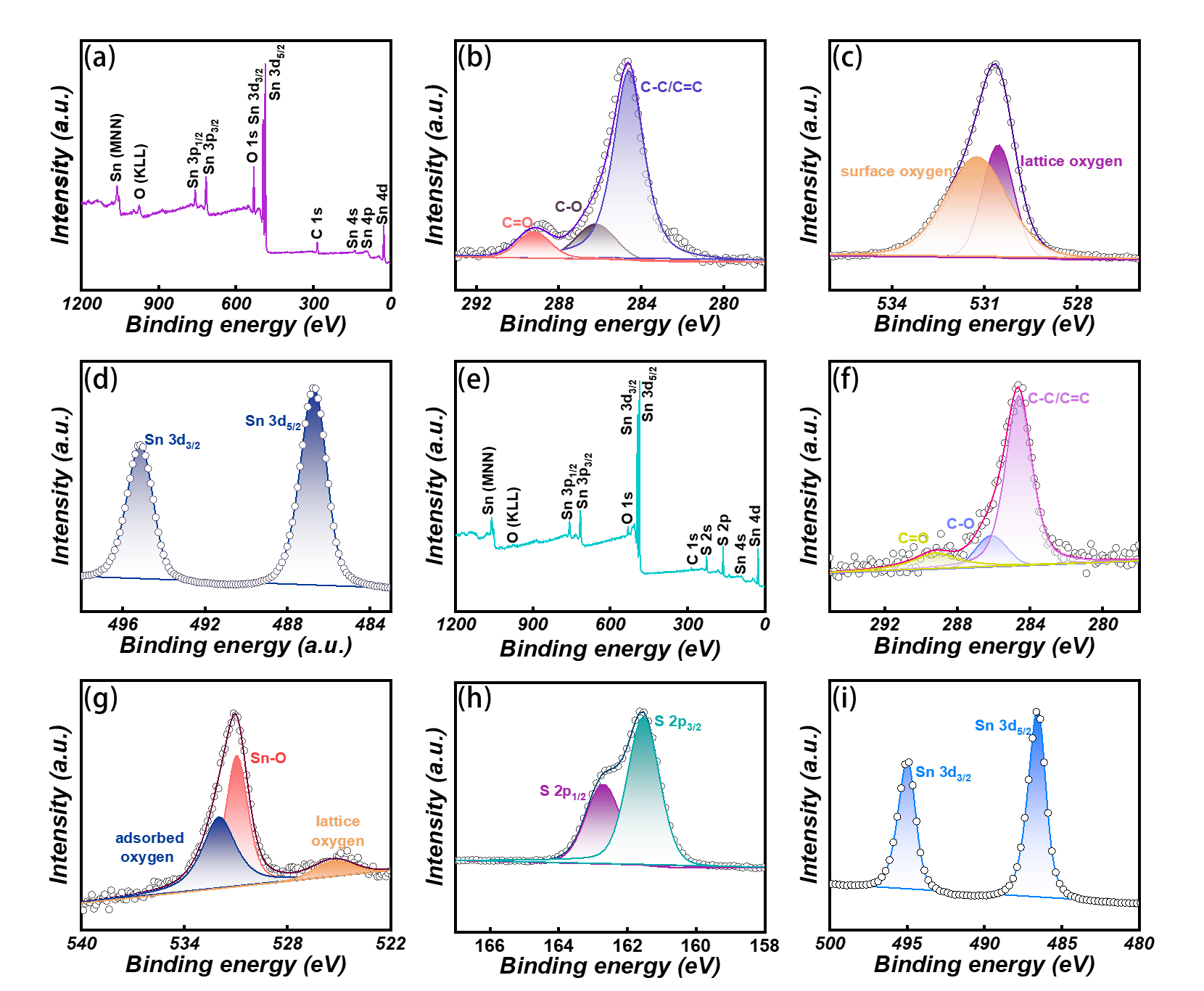


**Fig. S2** **a** XPS total spectrum, **b** C 1s, **c** O 1s, and **d** Sn 3d of SnO_2_/CF. **e** XPS total spectrum, **f** C 1s, **g** O 1s, **h** S 2p, and **i** Sn 3d of SnS_2_/SnO_2_/CF


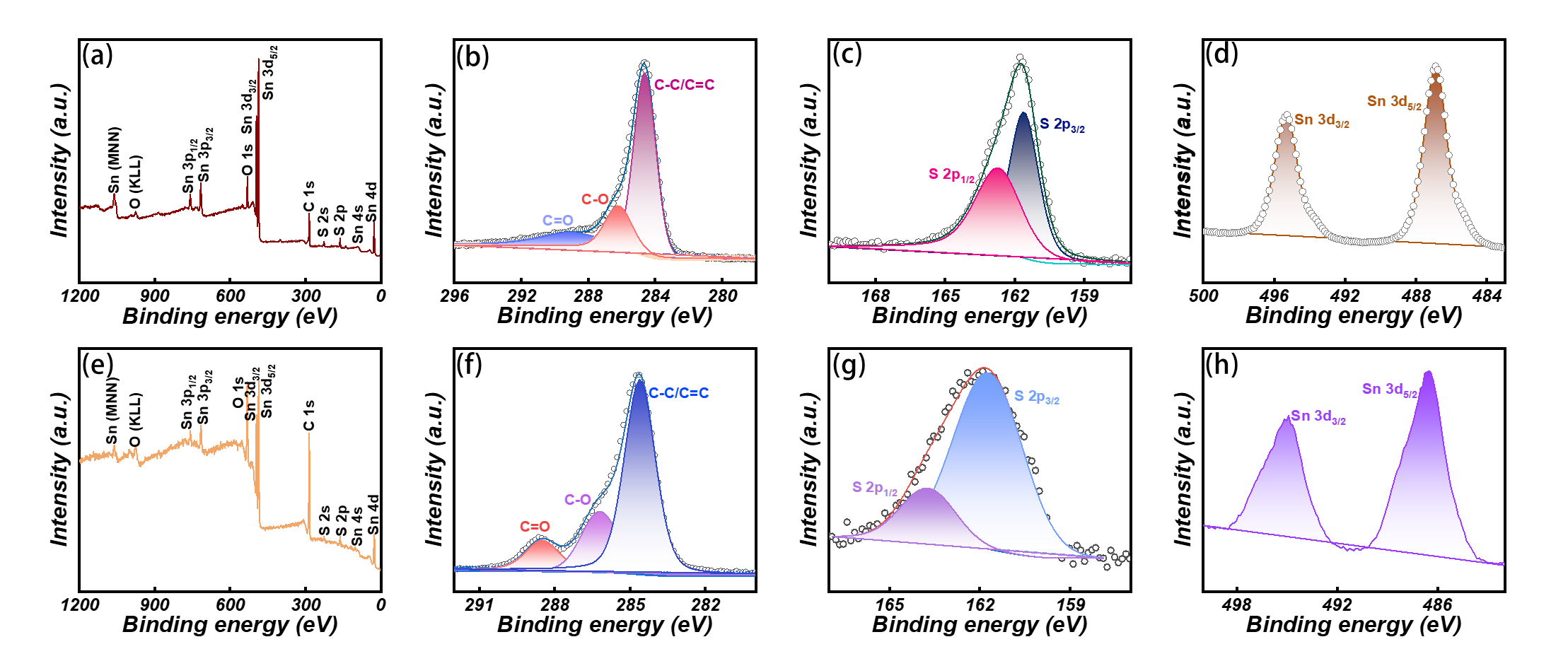


**Fig. S3 a** XPS total spectrum, **b** C 1s, **c** S 2p, and **d** Sn 3d of SnS/SnS_2_/CF. **e** XPS total spectrum, **f** C 1s, **g** S 2p, and **h** Sn 3d of SnS/CF


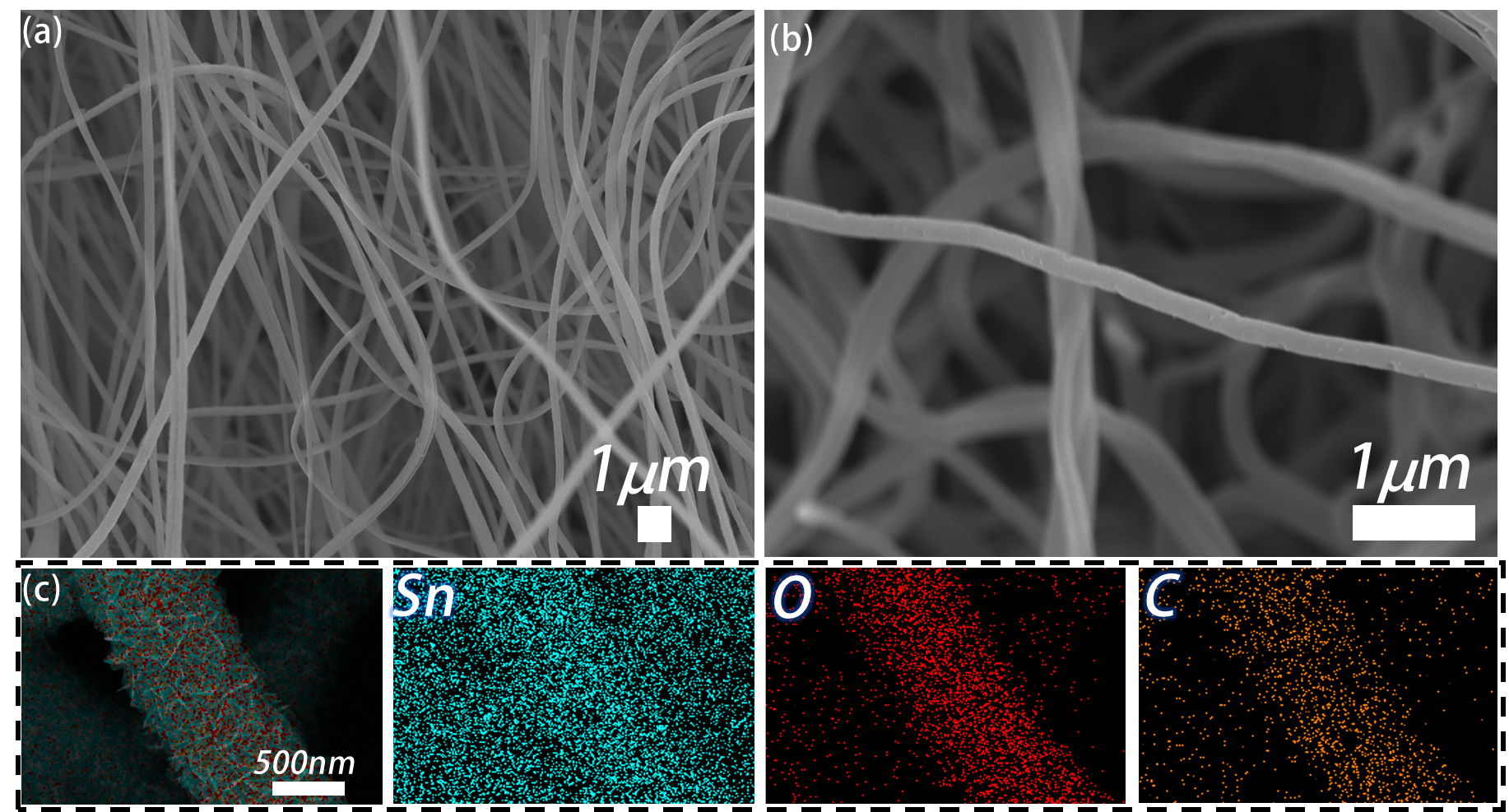


**Fig. S4** **a** SEM image of PANF. **b** SEM image of CF. **c** EDS elemental mapping image of SnO_2_/CF


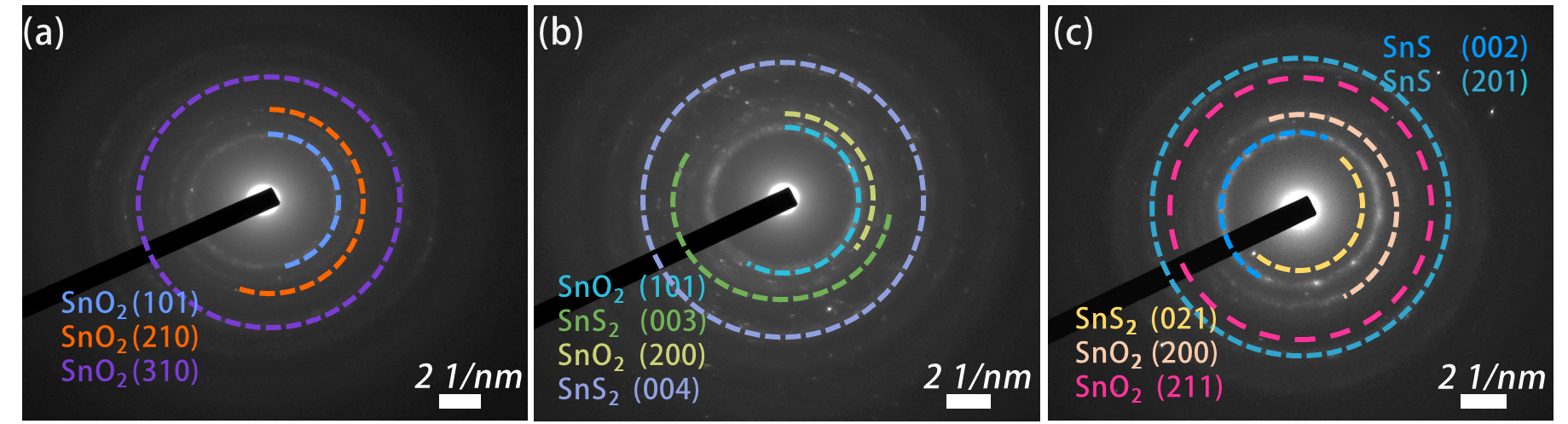


**Fig. S5** SAED image of **a** SnO_2_/CF, **b** SnS_2_/SnO_2_/CF, and **c** SnS/SnS_2_/SnO_2_/CF


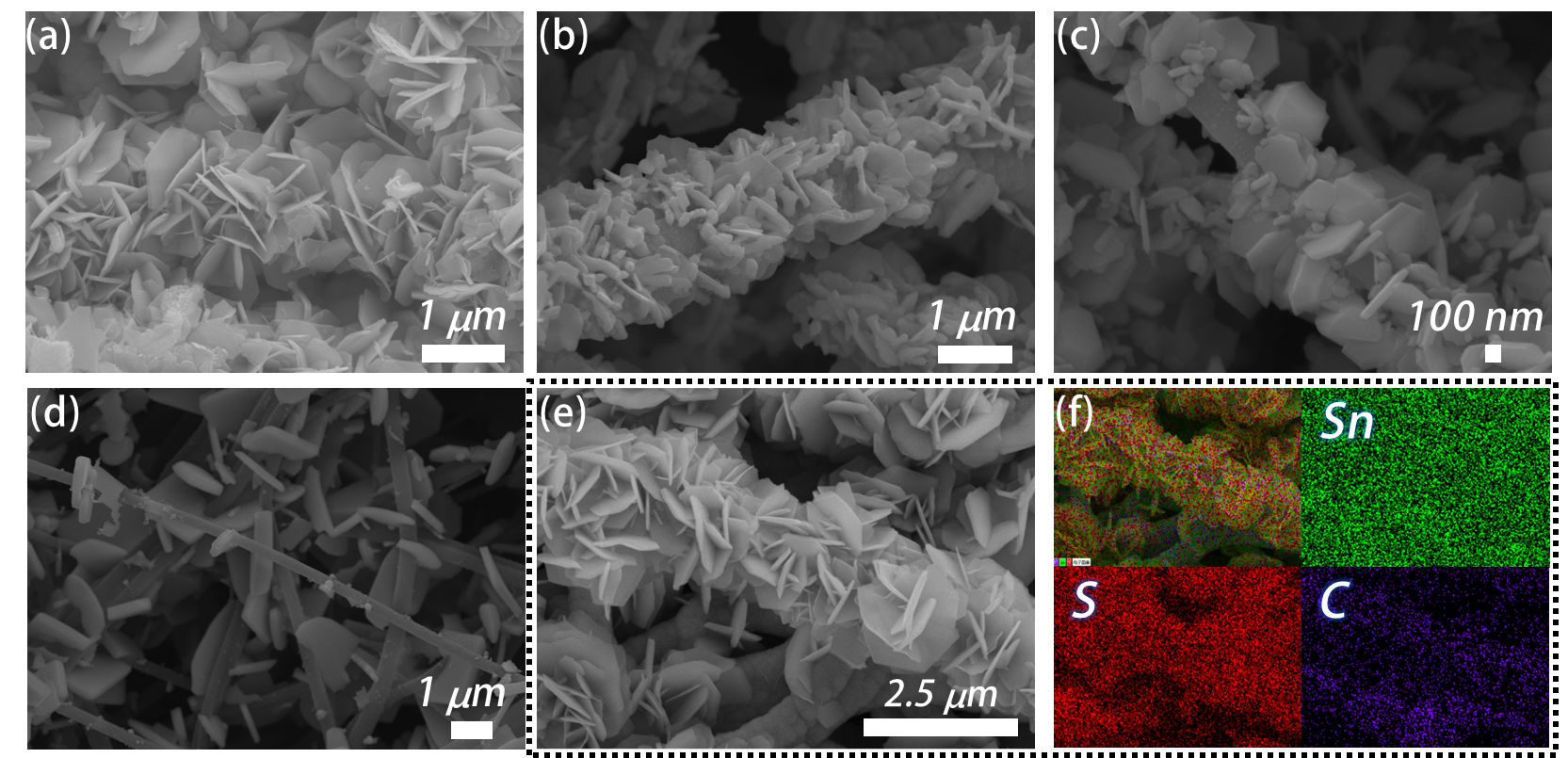


**Fig. S6** **a-d** SEM of SnS_2_/CF, SnS/SnS_2_/CF-0.25-410, SnS/SnS_2_/CF-0.25-450, and SnS/CF-0.25-500. **e, f** EDS elemental mapping image of SnS_2_/CF


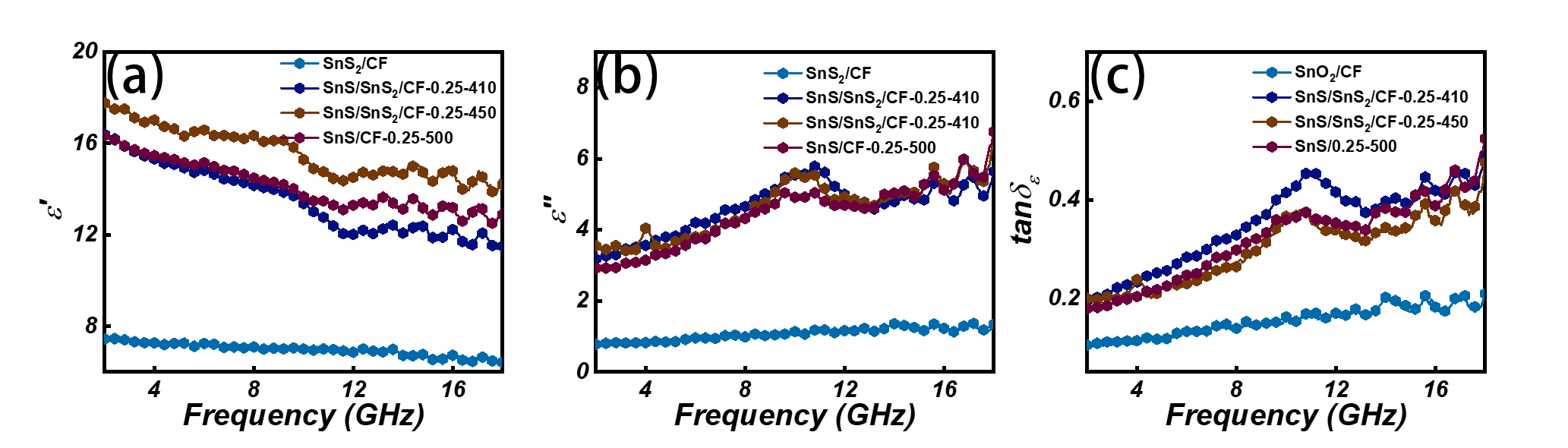


**Fig. S7** **a** *ɛʹ*, **b** *ɛ″*, **c** tan*𝛿_ɛ_*, of SnS_2_/CF, SnS/SnS_2_/CF-0.25-410, SnS/SnS_2_/CF-0.25-450, and SnS/CF-0.25-500


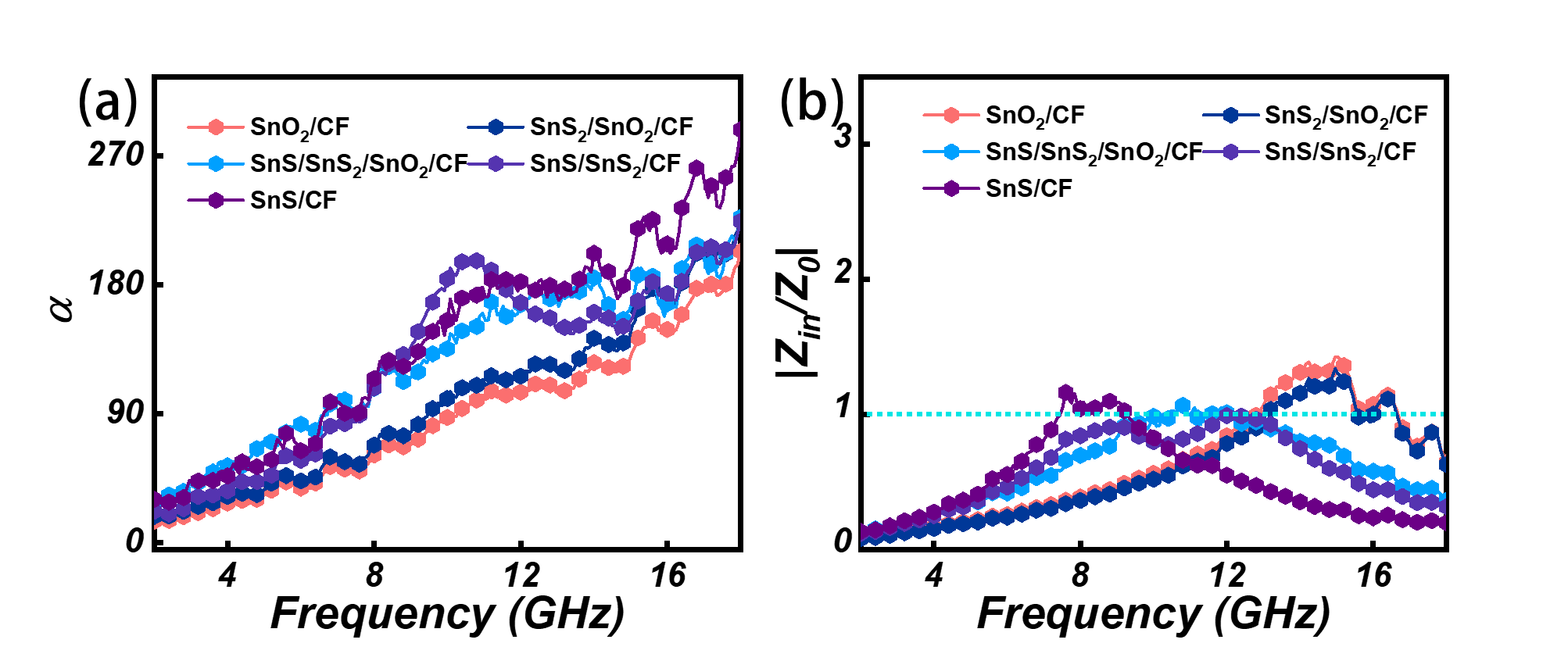


**Fig. S8** **a** *α*, **b** |*Z*_in_/*Z*_0_| of SnO_2_/CF, SnS_2_/SnO_2_/CF, SnS/SnS_2_/SnO_2_/CF, SnS/SnS_2_/CF, and SnS/CF


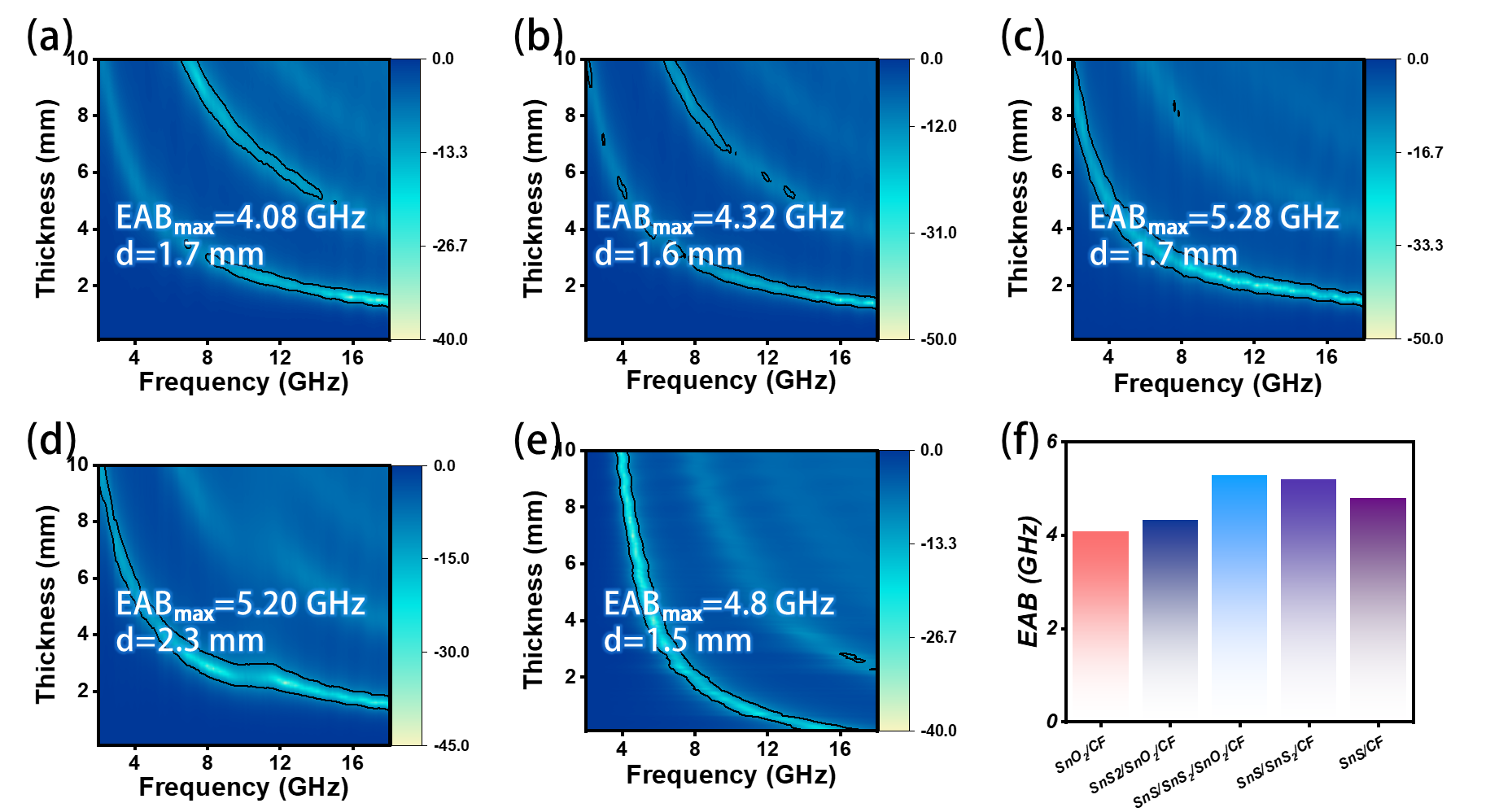


**Fig. S9** **a-f** 2D bandwidth map of SnO_2_/CF, SnS_2_/SnO_2_/CF, SnS/SnS_2_/SnO_2_/CF, SnS/SnS_2_/CF, and SnS/CF


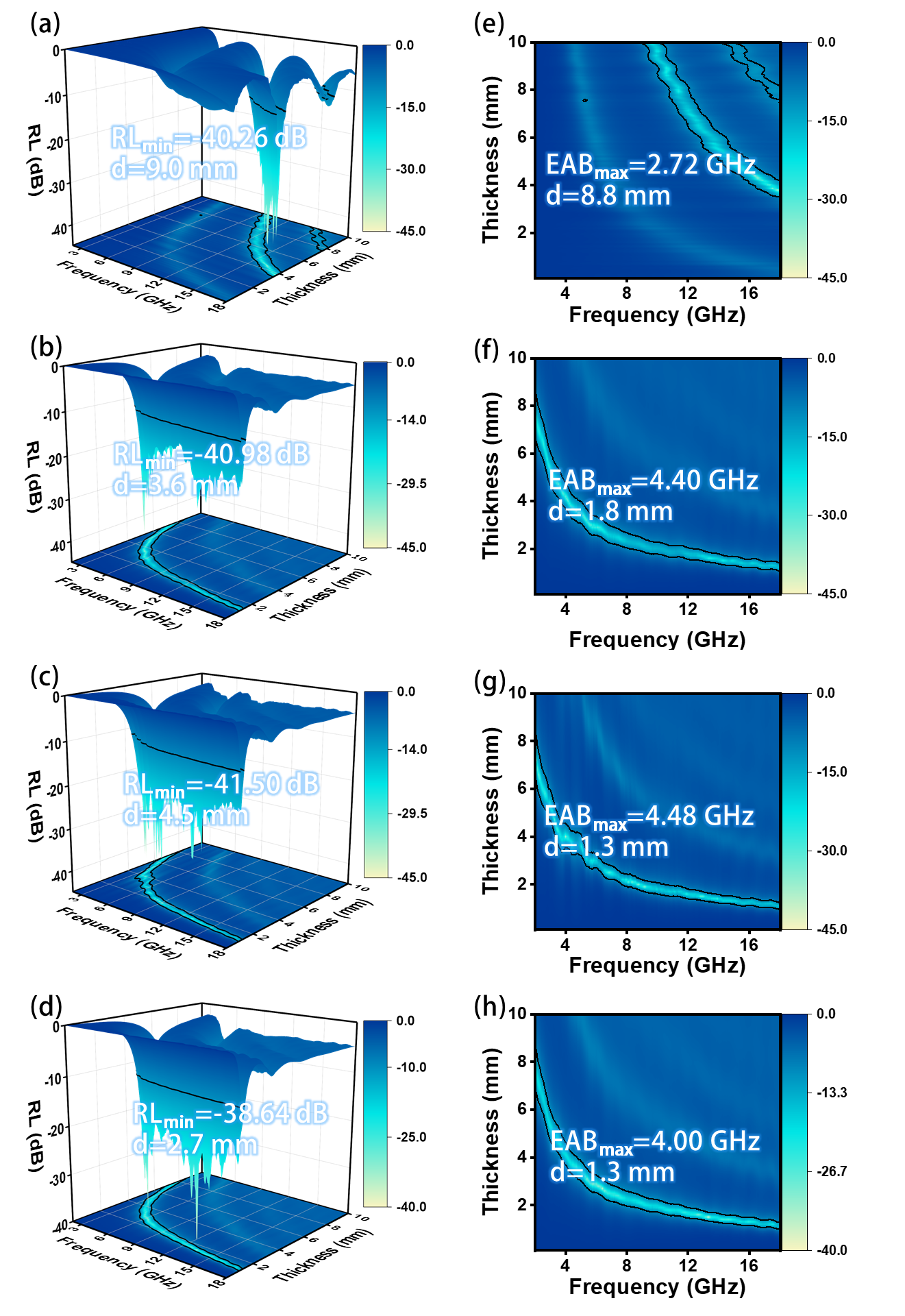


**Fig. S10 a-d** 3D RL plots and **e**-h 2D EAB plots of SnS_2_/CF, SnS/SnS_2_/CF-0.25-410, SnS/SnS_2_/CF-0.25-450, and SnS/CF-0.25-500


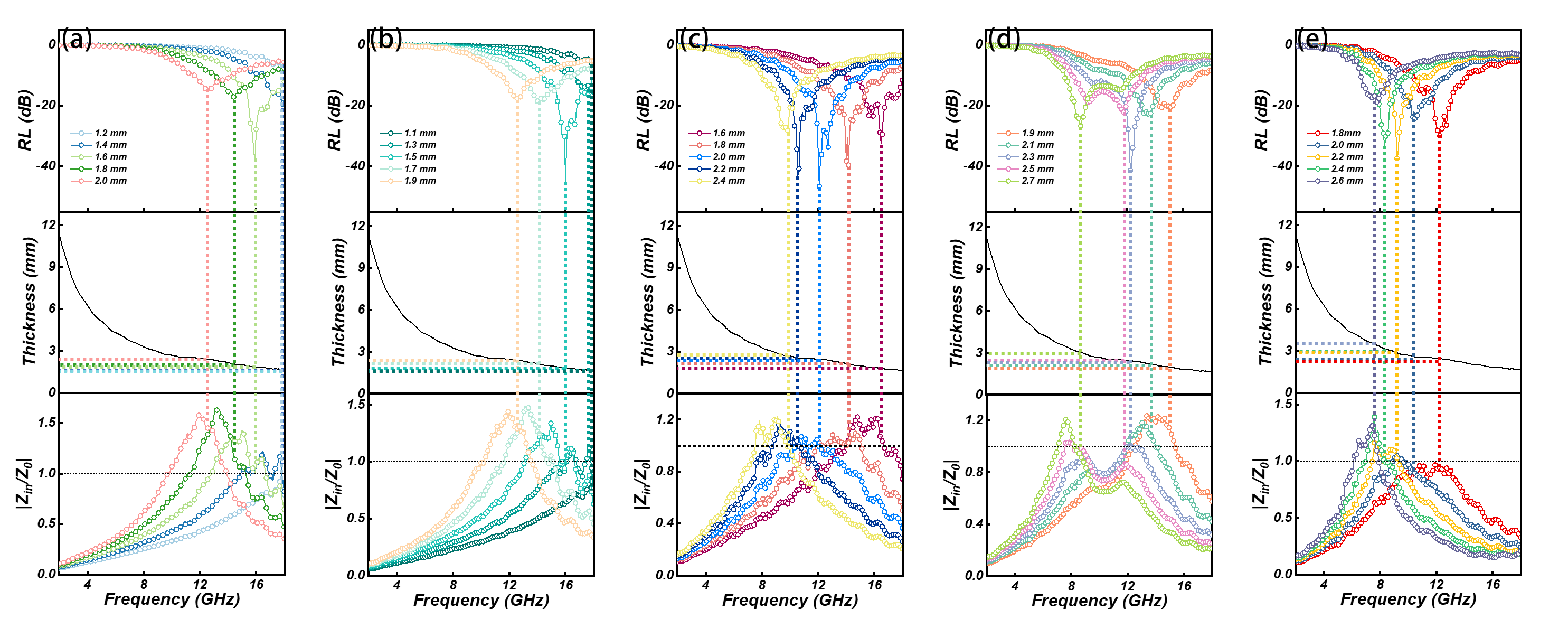


**Fig. S11** The dependence plot of *λ*/4 matching thickness vs RL peak vs |Z_in_/Z_0_| of the sample **a** SnO_2_/CF, **b** SnS_2_/SnO_2_/CF, **c** SnS/SnS_2_/SnO_2_/CF, **d** SnS/SnS_2_/CF, and **e** SnS/CF

**Fig. S12** Comparison SnS/SnS_2_/SnO_2_/CF and other works


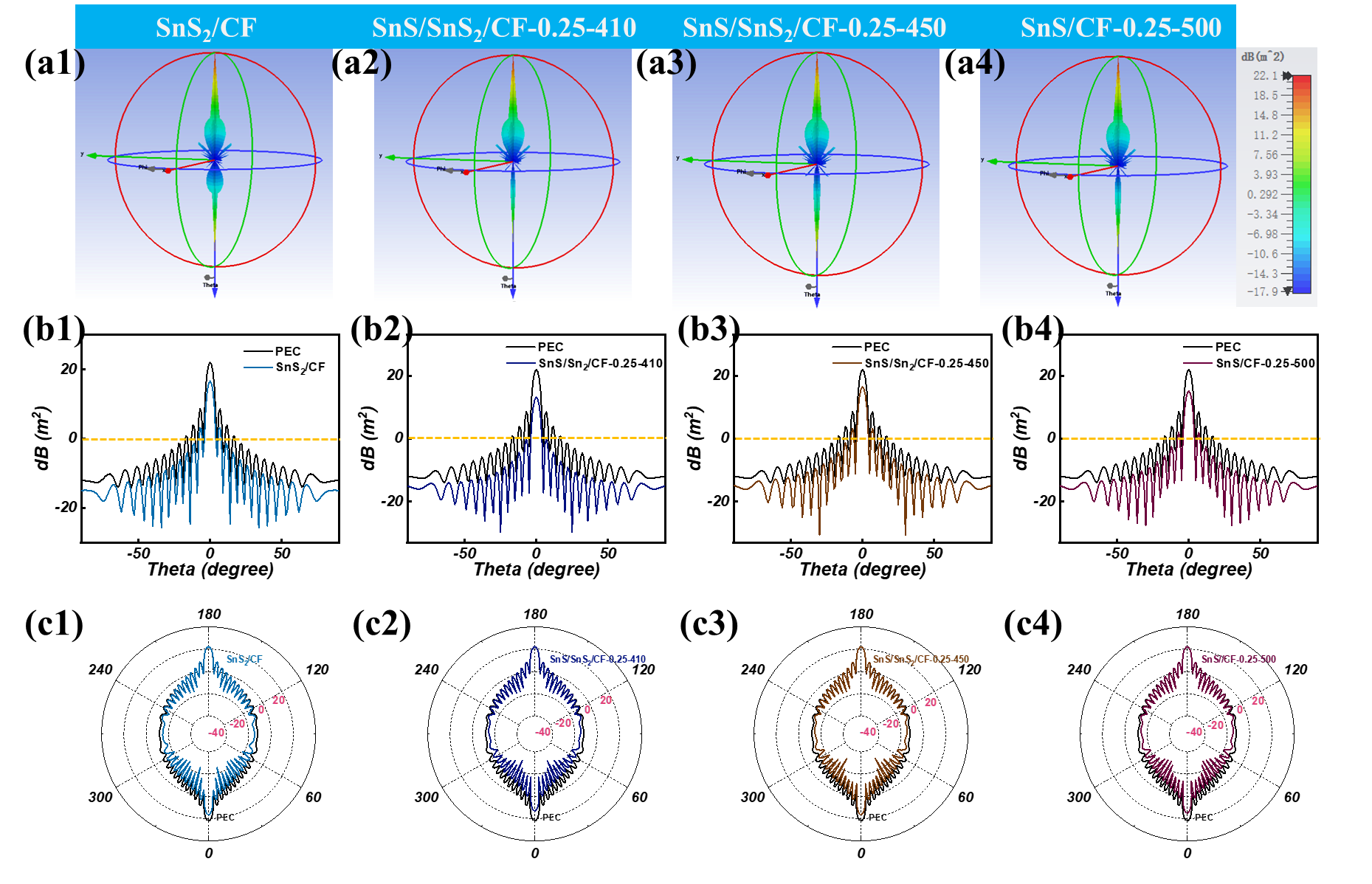


**Fig. S13 a** 3D radar wave scattering signals, **b** RCS simulation curves, and **c** RCS simulation results of polar coordinates of SnS_2_/CF, SnS/SnS_2_/CF-0.25-410, SnS/SnS_2_/CF-0.25-450, and SnS/CF-0.25-500


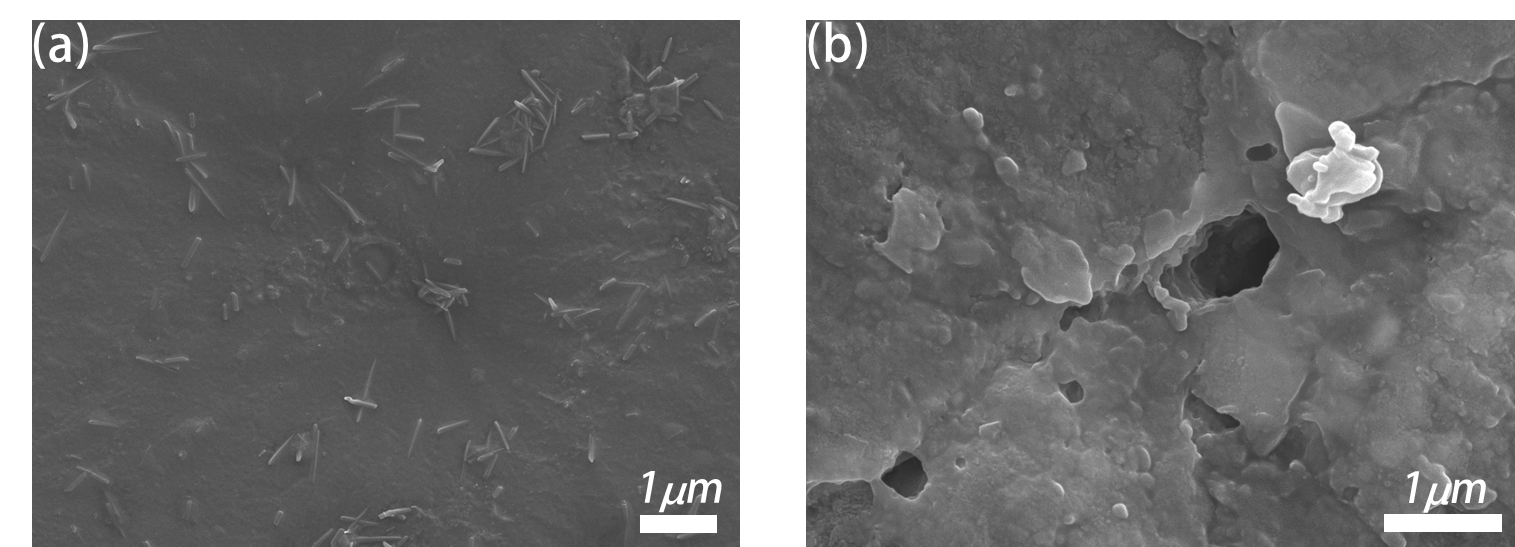


**Fig. S14** **a** SEM images of the coating of SnS/SnS_2_/SnO_2_/CF composite coating and **b** Pure epoxy coating after 14 days of immersion

**Table S1** Comparison SnS/SnS_2_/SnO_2_/CF and other works

| Sample | RL_min_/dB | EAB_max_/GHz | References |
| --- | --- | --- | --- |
| NiSn/CNFs | -39.81 | 5.24 | [S1] |
| Fe(acac)_3_/CF | -29.27 | 4.5 | [S2] |
| Sn-CNTs | -43.87 | 3 | [S3] |
| Fe-HPCNFs | -46.9 | 3 | [S4] |
| NiS_2_/SnS_2_ | -52.97 | 4.8 | [S5] |
| CF@Co_x_O_y_ | -19.2 | 2.6 | [S6] |
| Ni/SnO_2_ | -36.7 | 3.4 | [S7] |
| Fe/Fe_3_O_4/_C fibers | -40.1 | 3.26 | [S8] |
| SnO_2_@Fe_3_O_4_ | -20 | 5.6 | [S9] |
| SnO_2_(Ni_3_Sn_2_)@Ni_3_ | -37.4 | 5.28 | [S10] |

**Supplementary References**

[S1] W. Wang, M.H. Cao, Ni_3_Sn_2_ alloy nanocrystals encapsulated within electrospun carbon nanofibers for enhanced microwave absorption performance. Mater. Chem. Phys. **177**, 198e205 (2016). <http://dx.doi.org/10.1016/j.matchemphys.2016.04.018>

[S2] Y.H. Zhao, Z.C. Lou, Q.Y. Wang, Y.H. Wang, W. Sun et al., Thermal phase transition controlling electromagnetic wave absorption behavior of PAN fiber derived porous magnetic absorber. J. Mater. Sci-Mater. El. **32**(21), 26007-26020 (2021). <https://doi.org/10.1007/s10854-021-05864-z>

[S3] D.X. Wang, M.F. Saleem, M. Javid, X.H. Qu, A. Farid et al., Formation of Sn filled CNTs nanocomposite: Study of their magnetic, dielectric properties and enhanced microwave absorption performance at gigahertz frequencies. Ceram. Int. **48**, 21961-21971 (2022). <https://doi.org/10.1016/j.ceramint.2022.04.183>

[S4] R.L. Sun, G.L. Yan, X.L. Zhang, Z.Y. Li, J.Y. Chen et al., Fe-ZIF-derived hollow porous carbon nanofibers for electromagnetic wave absorption. Chem. Eng. J. **455**, 140608 (2023). <https://doi.org/10.1016/j.cej.2022.140608>

[S5] Y.Y. Dong, X.J. Zhu, F.Pan, Z. Xiang, X. Zhang et al., Fire-retardant and thermal insulating honeycomb-like NiS_2_/SnS_2_ nanosheets @ 3D porous carbon hybrids for high-efficiency electromagnetic wave absorption. Chem. Eng. J. **426**, 131272 (2021). <https://doi.org/10.1016/j.cej.2021.131272>

[S6] J.B. Chen, J. Zheng, Q.Q. Huang, G.H. Wang, G.B. Ji, Carbon fibers@Co-ZIFs derivations composites as highly efficient electromagnetic wave absorbers. J. Mater. Sci. Technol. **94**, 239-246 (2021). <https://doi.org/10.1016/j.jmst.2021.03.072>

[S7] B. Zhao, W.Y. Zhao, G. Shao, B.B. Fana, R. Zhang, Corrosive synthesis and enhanced electromagnetic absorption properties of hollow porous Ni/SnO_2_ hybrids. Dalton T. **44**, 15984 (2015). <https://doi.org/10.1039/c5dt02715b>

[S8] S.T. Yuan, T.C. Wang, T. Feng, J. Kong, Electromagnetic wave absorption of fabricated Fe/Fe3O4/C hollow fibers derived from ceiba fiber templates. Mater. Sci. Eng. B **299**, 117057 (2024). <https://doi.org/10.1016/j.mseb.2023.117057>

[S9] Z.H. Deng, S.R. He, W. Wang, M.Z. Xu, H.Y. Zheng et al., Construction of hierarchical SnO_2_@Fe_3_O_4_ nanostructures for efficient microwave absorption. J. Magn. Magn. Mater. **498**, 166224 (2020). https://doi.org/10.1016/j.jmmm.2019.166224

[S10] B. Zhao, X.Q. Guo, W.Y. Zhao, J.H. Deng, B.B. Fan et al., Facile synthesis of yolk-shell Ni@void@SnO_2_(Ni_3_Sn_2_) ternary composites via galvanic replacement/Kirkendall effect and their enhanced microwave absorption properties. Nano Res. **10**(1), 331-343 (2017). <https://doi.org/10.1007/s12274-016-1295-3>
